# Genome distribution of differential homoeologue contributions to leaf gene expression in bread wheat

**DOI:** 10.1111/pbi.12486

**Published:** 2015-10-07

**Authors:** Andrea L. Harper, Martin Trick, Zhesi He, Leah Clissold, Alison Fellgett, Simon Griffiths, Ian Bancroft

**Affiliations:** ^1^ Department of Biology University of York York UK; ^2^ John Innes Centre Norwich Research Park Norwich UK; ^3^ Present address: The Genome Analysis Centre Norwich Research Park Norwich NR4 7UH UK

**Keywords:** differential expression, bread wheat, genome dominance

## Abstract

Using a combination of *de novo* transcriptome assembly, a newly developed 9495‐marker transcriptome SNP genetic linkage map and comparative genomics approaches, we developed an ordered set of nonredundant transcripts for each of the subgenomes of hexaploid wheat: A (47 160 unigenes), B (59 663 unigenes) and D (40 588 unigenes). We used these as reference sequences against which to map Illumina mRNA‐Seq reads derived from young leaf tissue. Transcript abundance was quantified for each unigene. Using a three‐way reciprocal BLAST approach, 15 527 triplet sets of homoeologues (one from each genome) were identified. Differential expression (*P* < 0.05) was identified for 5248 unigenes, with 2906 represented at greater abundance than their two homoeologues and 2342 represented at lower abundance than their two homoeologues. Analysis of gene ontology terms revealed no biases between homoeologues. There was no evidence of genomewide dominance effects, rather the more highly transcribed individual genes were distributed throughout all three genomes. Transcriptome display tile plot, a visualization approach based on CMYK colour space, was developed and used to assess the genome for regions of skewed homoeologue transcript abundance. Extensive striation was revealed, indicative of many small regions of genome dominance (transcripts of homoeologues from one genome more abundant than the others) and many larger regions of genome repression (transcripts of homoeologues from one genome less abundant than the others).

## Introduction

The high incidence of polyploidy found in the history of many of our modern crops supports the theory that duplication of genetic material is a powerful facilitator of speciation via ecological diversification and adaptation. Polyploids also exhibit novel phenotypes and biosynthetic pathways (Kliebenstein, 2008; Schranz *et al*., 2011). Following genome doubling, many gene copies are rapidly lost (Scannell *et al*., 2007), although this process appears not to be completely random, with similar types of genes such as kinases and transcription factors (where stoichiometry of interactions might be important) more likely to be retained in duplicate (Blanc and Wolfe, 2004; Schnable *et al*., 2009; Seoighe and Gehring, 2004; Tian *et al*., 2005).

Several mechanisms have been proposed for enabling the retention of duplicate gene copies. Neofunctionalization (Hughes, 1994) occurs when one gene copy is diverted towards a new beneficial function. Alternatively, subfunctionalization (Force *et al*., 1999) may occur when normally multifunctional gene copies divide their functional workload. Another possibility is that one of the gene copies may become silenced and in this case also, not necessarily at random. The recent allopolyploid *Senecio cambrensis* exhibits different patterns of gene expression relative to the progenitor species, even when re‐synthesized (Hegarty *et al*., 2006). Alternatively, the expression patterns of nascent wheat exhibited genome expression‐level dominance (ELD) (Li *et al*., 2014) where the offspring resembled the expression patterns of one of the parents more than the other. These studies suggest that expression changes may occur rapidly and nonrandomly after hybridization and that they may have important functional significance.

Bread wheat (*Triticum aestivum*) is an interesting model for studying the evolution of gene expression across polyploid genome compartments. It is an allohexaploid comprising three genomes: A, B and D, derived from multiple hybridization events between its diploid and tetraploid ancestors (Chantret *et al*., 2005). The first hybridization is thought to have occurred around 5.5 million years ago, between the ancestors of the A and B genome lineages leading to the formation of the D genome lineage by homoploid hybrid speciation (i.e. hybridization without change in chromosome number). Less than 800 000 years ago, a hybridization between the A genome progenitor, *Triticum urartu*, and the B genome progenitor, thought to be a close relative of *Aegilops speltoides*, formed the AABB allotetraploid emmer wheat *Triticum turgidum*. Finally, <400 000 years ago, hybridization between emmer wheat and the D genome progenitor, *Aegilops tauschii* (Marcussen *et al*., 2014), formed the AABBDD allohexaploid bread wheat, *T. aestivum*.

We hypothesize that some regions of the hexaploid wheat genome may differ from the expected equal contributions for homoeologues to the transcriptome, and aimed to test this. To analyse the spatial patterns of gene expression for each gene across the three genomes of bread wheat, we developed two resources using methodology adapted from previous work in *Brassica napus* (Bancroft *et al*., 2011; Harper *et al*., 2012; Higgins *et al*., 2012; Trick *et al*., 2009b). Although a chromosome‐based draft sequence for wheat is now available (International Wheat Genome Sequencing, 2014), the numerous small contigs produced by whole‐genome and chromosome‐shotgun sequencing of large repetitive genomes can lead to underrepresentation and mis‐assembly of repetitive sequences, as well as collapsed assemblies of related genes both within and (especially) between genomes. On the other hand, transcriptome assemblies have the benefit of reducing the genome complexity by focusing on only the expressed genes and the independent assembly of transcripts from each of the three progenitor species ensures the correct allocation of gene assembly to genome. In addition, *de novo* assembled transcripts are not prone to modelling errors of gene prediction programmes and matching of tissue and developmental stage of mRNA used for construction of the reference sequence with that used for the analysis ensures its suitability for the experimental design. For these reasons, we decided to develop a new transcriptome reference capable of discriminating between homoeologous transcripts, created by *de novo* assembly of transcripts from the diploid progenitor species, which were then improved using the tetraploid progenitor to ‘cure’ the B genome reference. We then developed a set of pseudomolecules to infer the order of the reference genes within the genome by developing a high‐density gene‐based genetic linkage map and exploiting the conserved synteny between wheat genomes and that of *Brachypodium distachyon*, which has been sequenced to a very high standard and shared a common ancestor with wheat 32–39 million years ago (International Brachypodium, 2010). The high degree of conserved synteny between the genomes of grasses (Moore *et al*., 1995) and the availability of a high‐quality genome sequence provides the opportunity to use comparative genomics to infer gene order based on that of orthologues in *B. distachyon*. Indeed, such cross‐species inference has already been used in estimation of genome organization in barley (International Barley Genome Sequencing *et al*., 2012; Mayer *et al*., 2011). By focusing on the analysis of transcribed sequences (mRNA‐Seq), we aimed to bypass the difficulties associated with genome sequence assembly in bread wheat, such as the large proportion of repetitive sequences between genes (Choulet *et al*., 2010) and the hexaploid nature of the species.

## Results

### Reference assembly

The underpinning resource for robust SNP‐calling and transcript quantification by RNA‐Seq is a reference sequence comprising nonredundant assemblies of transcribed sequences, *that is* unigenes. The first step was to assemble RNA‐Seq reads into unigenes representing each of the three progenitor genomes of bread wheat. We generated Illumina 100‐base paired‐end reads from mRNA isolated from young leaf tissue of *T. urartu*,* A. speltoides* and *A. tauschii* (representing the A, B and D genomes, respectively) and assembled sets of unigenes using the Trinity package (Grabherr *et al*., 2011). As the B genome in hexaploid wheat is much more closely related to that in tetraploid wheat, *T. turgidum* ssp.* dicoccoides*, the B genome unigenes were ‘cured’ (Higgins *et al*., 2012) to more closely represent those of bread wheat, using Illumina RNA‐Seq data from that tetraploid. The result was a reference transcriptome sequence for hexaploid wheat comprising 105 069, 132 363 and 85 296 unigenes representing its A, B and D genomes, respectively. For each unigene, the gene model with the greatest sequence similarity was identified in each of *B. distachyon* (Brachypodium), rice, Sorghum and Arabidopsis, and annotation extracted for the Brachypodium, rice and Arabidopsis models, as listed in Data S1.

### Linkage map construction

To support the *in silico* rearrangement of the Brachypodium genome to represent that of hexaploid wheat, we first constructed a high‐density unigene‐based SNP linkage map. This involved generating Illumina reads from mRNA isolated from young leaf tissue of 47 lines of a single‐seed descent linkage mapping population (http://www.wgin.org.uk) and the parents of the population (cultivars Paragon and Chinese Spring), then using them to simultaneously identify and score polymorphisms using the unigenes as reference sequences (Trick *et al*., 2009a,b). The scoring strings were used, in conjunction with the hypothetical fine‐scale order of the unigenes in which the polymorphisms were identified (based on sequence similarity to Brachypodium gene models), to construct a linkage map of hexaploid wheat based on recombination bins. The linkage map, for which details are provided in Data S2, contained 9495 markers, with fewer in the less polymorphic D genome (981) compared with the more polymorphic A and B genomes (4021 and 4493, respectively). The map established 624 consensus recombination bins, as shown in Data S3, and defining in detail collinearity with the Brachypodium genome, as shown in Data S4.

### Brachypodium‐based pseudomolecule assembly

**Figure 1:**
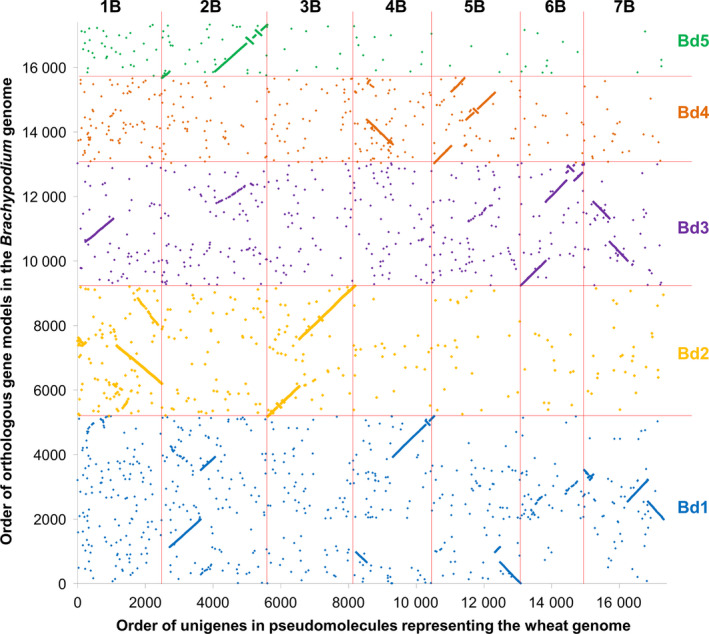
Collinearity between inferred gene order in wheat and Brachypodium. The plot shows the order in each genome of 17 315 wheat unigenes and their Brachypodium orthologues with points colour‐coded by sequence similarity to the chromosome assignment of Brachypodium gene models: blue for chromosome 1, orange for chromosome 2, purple for chromosome 3, brown for chromosome 4 and green for chromosome 5.

**Figure 2:**
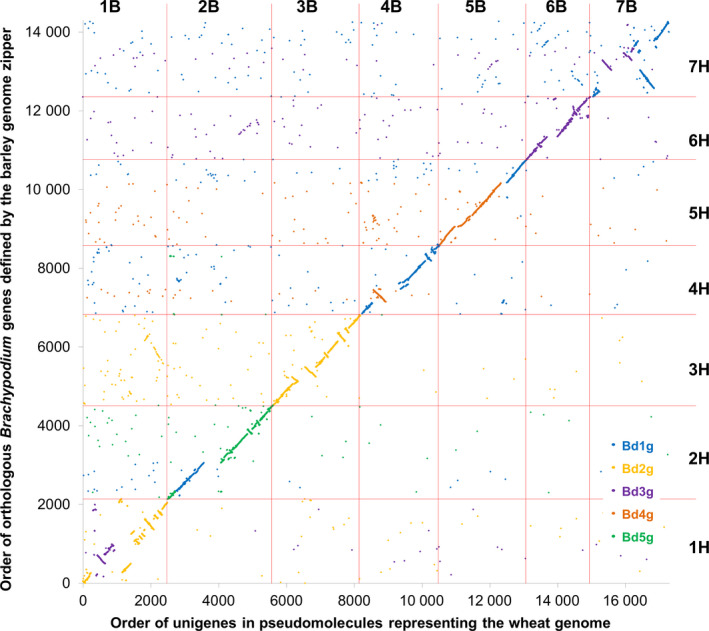
Collinearity between inferred gene order in wheat and barley. The plot shows the order in each genome of 8187 wheat unigenes and their barley orthologues with points colour‐coded by sequence similarity to the chromosome assignment of Brachypodium gene models: blue for chromosome 1, orange for chromosome 2, purple for chromosome 3, brown for chromosome 4 and green for chromosome 5.

Based on the ranges of Brachypodium gene models identified in each of 56 collinearity blocks, the Brachypodium genome sequence was rearranged *in silico* to establish a new order of Brachypodium genome sequences, to which we refer hereafter as pseudomolecules, representative of the organization of orthologous sequences in the wheat genomes, according to the design specified in Data S5. Finally, the positions of the assembled A, B and D unigenes on these pseudomolecules were established by sequence similarity. The resulting resource comprised 175 103 hypothetically ordered unigenes (58 320, 67 829 and 48 954 for the A, B and D genomes, respectively), as shown in Data S6. As some of these represented alternative splice forms, redundancy was reduced further by selecting the longest unigene where multiple unigenes mapped to the same location in the genome, resulting in a final transcriptome reference sequence for mapping sequence reads of 147 411 unigenes (47 160 for the A genome, 59 663 for the B genome and 40 588 for the D genome). We compared, as an example, the order of 17 315 unigenes along the B genome with the order in the Brachypodium genome of gene models with the greatest sequence similarity. The result provides a detailed gene‐based analysis of collinearity of the genome representations (Figure 1). We also compared, as an example, the order of 8187 unigenes along the B genome with the order inferred in the barley (*Hordeum vulgare*) genome (Mayer *et al*., 2012) for orthologues of the corresponding Brachypodium gene models. The result showed good overall collinearity between the wheat and barley genomes, but in detail the genomes are differentiated by numerous rearrangements (Figure 2). For completeness, we also compared the order of 21 483 unigenes against the wheat chromosome 3B annotated pseudomolecule (Figure S1) and the V5 wheat genome zipper (Figure S2) (both downloadable from http://wheat-urgi.versailles.inra.fr), which also showed extensive, but imperfect, collinearity.

### Visualization of regional unbalanced expression

To assess genome ELD patterns, 15 527 triplets of homoeologues were identified via a three‐way reciprocal BLASTn analysis (threshold *E*‐value 1E‐30). Although the reciprocal blast parameters are particularly stringent to exclude as many spurious homoeologous triplets as possible from the analysis, this is the largest panel of candidate homoeologous genes identified in hexaploid wheat to date.

Using the 147 411‐unigene transcriptome reference sequence, mRNA‐Seq reads from juvenile leaves of 54 bread wheat accessions were mapped. Transcript abundance was quantified and normalized as reads per kb per million aligned reads (RPKM) and the values extracted for the 15 527 homoeologue triplets.

**Figure 3:**
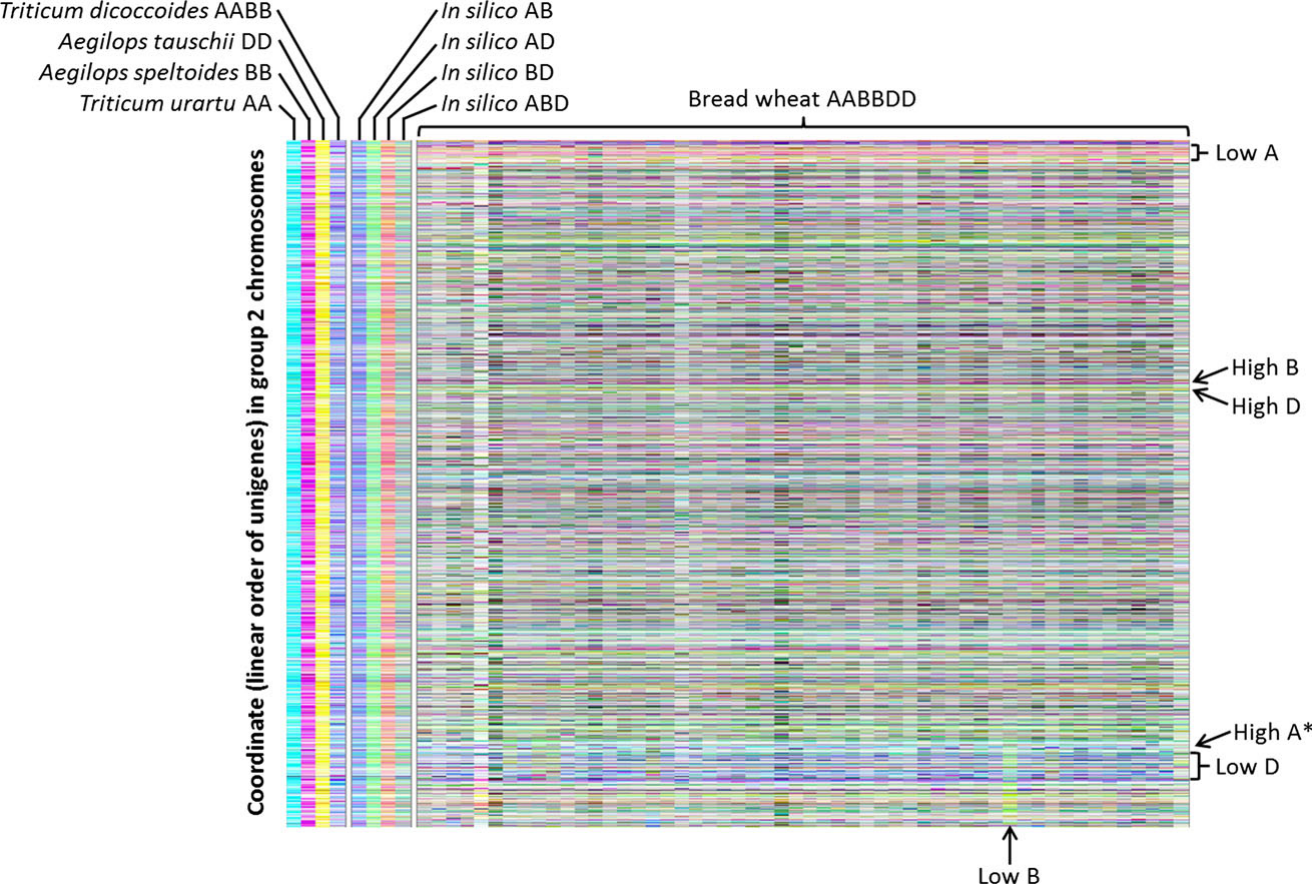
Tile plots illustrate relative transcript contributions for the A, B and D copies of 2571 triplets of homoeologous genes on linkage group 2. Represented are 54 bread wheat accessions, diploid ancestors *Triticum urartu* (AA), *Aegilops speltoides* (BB) and *Aegilops tauschii* (DD), tetraploid ancestor *Triticum dicoccoides* (AABB), and *in silico* tetra‐ and hexaploid combinations. The A genome is represented by cyan, B genome magenta and D genome yellow. The homoeologous genes are arranged in pseudomolecule order (which is largely identical for all three genomes). Regions of interest are marked, including the region used for validation (*).

We developed a visualization method based on assigning quantitative transcript abundance (RPKM) for each member of the 15 527 homoeologue triplets a value in CMYK colour space where the contributions from the A, B and D genome copies were coded to cyan, magenta and yellow channels, respectively, and displaying the results using tile plots. We termed the method transcriptome display tile plot. As controls, and to provide a visual key, mRNA‐Seq reads (down‐sampled to 33 million reads) from juvenile leaves of each of the diploid wheat species were mapped onto the unigene reference sequence (incorporating all three genomes) and the relative expression across the homoeologous triplets visualized, as shown in Figures 3 and S3. Although there is slight colour distortion from cross‐mapping of reads to alternate homoeologues of some triplets (the expected consequence of stretches of identical sequences of ~100 bases shared by homoeologous genes), the signals are predominantly as expected: cyan for *T. urartu*, magenta for *A. speltoides* and yellow for *A. tauschii*. Using the mRNA‐Seq reads from combinations of these diploids to simulate the visualization of polyploids, these *in silico* combinations generated, with some colour distortion arising from cross‐mapping, the expected predominant signals: blue for A plus B genomes, green for A plus D genomes, red for B plus D genomes and grey for A plus B plus D genomes. As a final control, mRNA‐Seq reads for *T. turgidum* (the AABB allotetraploid) were mapped, producing the expected predominantly blue signal.

### Comparison with genome sequence data

The 15 527 triplet sequences were compared to the complete panel of high‐confidence gene models (not including splice variants), and 8605 homoeologous triplets identified from genome sequence data (International Wheat Genome Sequencing Consortium, 2014; Pfeifer *et al*., 2014). By comparing our triplet panel to the IWGSC A, B and D gene models (totalling 32 081, 34 226 and 33 079 models, respectively), our models showed high similarity with 54.5%, 50.4% and 51.2% of IWGSC A, B and D gene models, respectively. When we compared our triplets to the IWGSC triplets, we matched 76.8%, 76.2%, 76.0% of genes from the A, B and D genome, respectively. Conversely, when the IWGSC triplets were compared to our set, they only matched 48.8%, 46.0% and 45.6% of our genes, respectively.

These results suggest that as well as identifying the majority of triplets that had been found in previous studies, this approach was able to detect roughly double the total number of candidate homoeologous triplets. However, as the estimated number of genes on each of the subgenomes is likely to be between 28 000 and 36 000 (Brenchley *et al*., 2012; Choulet *et al*., 2010; Hernandez *et al*., 2012; Massa *et al*., 2011), it is likely that there are many triplets that could not be identified using such a stringent method. Consistent with this, we found that around half of the total IWGSC gene models were represented in our triplet panel.

### Assessment of genome expression‐level dominance

A Tukey test was used to identify homoeologues with transcripts from one copy varying in abundance from the other two. A total of 5248 unigenes showed significant differential expression from one of the genomes (*P* < 0.05) and were thus assigned as up‐ or down‐regulated (Figure S4). Of these, 1074, 978 and 854 A, B and D homoeologues, respectively, were found to be up‐regulated, and 574, 1132 and 636 A, B and D homoeologues were found to be down‐regulated (Data S7). These unigenes exhibited some localized clustering, with those clusters distributed throughout the genome.

### Validation of differential expression of homoeologues

The method typically used for validating gene expression differences inferred from mRNA‐Seq data is quantitative reverse transcription PCR (qRT‐PCR). This method is, however, slow and expensive, and in polyploid genomes, assays frequently cannot be designed. We therefore developed a method to quantify the *relative* contributions to the mRNA pool of each homoeologue based on the relative proportions in derived cDNA of each base at interhomoeologue polymorphisms (IHPs). As an example, the A, B and D transcript assemblies A_comp34236_c0_seq1, B_comp4776_c0_seq1 and D_comp10830_c0_seq1, the products of which are so far uncharacterized, were one of the homoeologue triplets exhibiting significant genome expression imbalance identified using Tukey tests (Figure S4), in this case with the A genome copy being 3.5‐fold higher on average than the B genome homoeologue, and fourfold higher than the D genome homoeologue (Figure S5). These three reference sequences were aligned (Figure S6), and putative IHPs identified and genome assigned depending on the genome showing the alternative base. A set of primers was then designed to amplify and Sanger sequence all three transcripts simultaneously from cDNA. All predicted IHP positions could be confirmed as they appeared as double peaks in the subsequent sequence traces (Figure S7). As the PCR was performed on cDNA, these cDNA‐based polymorphisms exhibit peak height variation proportional to the relative abundance in the RNA samples of the transcripts of the underlying genes. At any given IHP, peak height ratios were calculated as polymorphic base to ancestral base using Softgenetics Mutation Surveyor software (State College, PA, USA). The null hypothesis (no expression dominance) would be for the A‐, B‐ and D‐linked IHP variant bases to have roughly equal peak height ratios. However, consistent with the mRNA‐Seq data, we found that the A genome ratios were approximately 4.7‐fold higher than the B and fivefold higher than the D peak height ratios, confirming the mRNA‐Seq‐based calling of differential expression (Data S8).

## Discussion

As an allohexaploid with several hybridization events spread across the last 5 million years of its history, bread wheat is an interesting model for studying the evolution of gene expression following the sudden increases in transcript levels that must have occurred after each event. We hypothesized that the genomes would not contribute equally to the pool of transcripts, and consistent with this, we found extensive variation in the relative expression of triplets of homoeologues in the tissue analysed (leaves). On the whole, no one genome dominated the mRNA pool. In fact, the imbalances appeared to be largely random from gene to gene, with no functional bias identified by analysis of gene ontology (data not shown). Some small regions did show distinct banding patterns, however, suggesting regional genome dominance (yellow, cyan and magenta colours, as illustrated in Figures 3 and S3). More prominent are the larger regions for which one genome appears to have been repressed (red, green and blue colours, as illustrated in Figures 3 and S3). A rationale for such regions would be the suppression of one or more genes with detrimental effects. Both genome dominance and genome repression effects are remarkably consistent across the panel of 54 varieties of bread wheat analysed.

These results are consistent with indications from previous studies which found signs of genome asymmetry amongst neighbouring genes (Pfeifer *et al*., 2014) and a lack of global genome dominance (International Wheat Genome Sequencing Consortium, 2014; Pfeifer *et al*., 2014). In addition, there were just a few regions, all large, for which imbalances can be observed in individual (or just a few) varieties, which indicate specific long‐range silencing or perhaps genome deletion/homoeologous exchange events as have been observed in other polyploid crops, such as oilseed rape (Chalhoub *et al*., 2014).

We were able to study the relative contributions of each genome to the total pool of transcripts in bread wheat through the development of a set of resources based on mRNA‐Seq. As the cost of sequencing declines, genotyping by sequencing (GBS) approaches are becoming ever more popular for genetic analyses. The focus for reduced representation methods (to reduce complexity, and hence costs) has been firmly on genome‐targeted approaches such as RAD‐Seq, which generates sequences adjacent to restriction endonuclease cleavage sites and has been applied successfully in plants (Baird *et al*., 2008; Elshire *et al*., 2011; Miller *et al*., 2007) including for linkage map construction (Chutimanitsakun *et al*., 2011; Poland *et al*., 2012). There are important limitations to the approach, particularly for species with large, complex genomes such as that of bread wheat. The ordering of markers, for example by alignment to a genome sequence resource, is necessary to realize the full power of approaches such as genomewide association scans, as a cluster of markers in LD with a locus controlling variation for a trait provides more compelling evidence than single‐marker associations. This ordering can be difficult for RAD‐Seq markers, for example, as the majority of polymorphisms identified will be in noncoding sequences, which necessitates the use of the genome sequence of the species being studied. In contrast, focusing on transcribed sequences, by mRNA‐Seq, enabled us to fully exploit comparative genomics, by utilizing collinearity of coding regions between wheat and Brachypodium. In more generalized applications of the approach, even relatively distantly related orthologous genes can readily be identified, although conservation of synteny can be expected to decrease with increasing genetic distance. Furthermore, mRNA‐Seq data can be analysed for SNP variation as well as transcript abundance, both of which can then be used in association genetic approaches such as associative transcriptomics (Harper *et al*., 2012).

## Experimental procedures

### Growth of materials

*Triticum urartu*,* A. speltoides*,* A. tauschii* and *T. turgidum dicoccoides*, 47 lines of a single‐seed descent linkage mapping population derived from a Chinese Spring × Paragon cross and 54 lines from a diversity panel of *T. aestivum* plants were grown for transcriptome sequencing and validation. The seeds were placed on moist filter paper and placed in a refrigerator at 6 °C for 2 days before being transferred to a germinator at 20 °C overnight. Seeds were then transferred to pots with a peak/sand mix and arranged in four‐block, one‐way randomized design with one plant of each of the accessions per block and randomized within each block. Plants were grown in long‐day glasshouse conditions (16‐h photoperiod) at 15 °C (400W HQI metal halide lamps).

### RNA extraction and cDNA synthesis

Second true leaves from each of four plant replicates per accession were harvested approximately 14 days after pricking out (21 days after sowing) as close to the mid‐point of the light period as possible, pooled and immediately frozen in liquid nitrogen. Samples were extracted using the Omega Biotek EZNA Plant RNA Kit (Norcross, GA, USA) according to the manufacturer's instructions. cDNA was synthesized using standard protocols from 2 μL of total RNA.

### Leaf transcriptome sequencing

Illumina sequencing, quality control and data processing were conducted as described previously (Bancroft *et al*., 2011). The HiSeq2500 platform was used to generate 100‐base, paired‐end reads for the progenitor lines and single‐end reads for the mapping lines and diversity panel.

### Development of Brachypodium‐based pseudomolecules

First, using Trinity r2012‐03‐17 (Grabherr *et al*., 2011), transcriptome assemblies were constructed separately for each of the diploid species, *T. urartu* (101 million reads), *A. speltoides* (124 million reads) and *A. tauschii* (98 million reads), yielding 123 236 A assemblies, 169 009 B assemblies and 98 063 D assemblies, respectively. Redundancy within each set was then removed with CD‐HIT v4.5.4 (Li and Godzik, 2006) using an identity threshold of 0.95 and a word length of 5. This step reduced the assemblies to 105 069, 132 363 and 85 296 representing A, B and D clusters, respectively. The sequence identifiers generated by Trinity were prepended with their genome of origin in order to distinguish them and the three FASTA files were then simply combined to produce a transcriptome of 322 728 unigenes. When this was used as a reference sequence for alignment of hexaploid Chinese Spring RNA‐Seq reads, it was found that a relatively low number of reads mapped to the B genome assemblies, suggesting that *A. speltoides* was not an ideal B genome proxy. The B genome assemblies within this set were therefore ‘cured’ using reads obtained from the tetraploid *T. turgidum dicoccoides*, which contains a B genome more closely related to that in hexaploid wheat, using a method previously described (Higgins *et al*., 2012). Curing refers to the correction of reference sequences by repeated rounds of alignment and adjustment. Reads were aligned to the uncured reference and mismatched sites corrected in the B genome reference. After six cycles of curing, a total of 198 607 bases over the B genome assemblies had been modified. The edited file was then used as the reference for alignment with reads for transcript quantification. The unigenes were aligned, using BLASTn (Altschul *et al*., 1990) with an *E*‐value threshold of 1E‐30, against annotated gene sets for *B. distachyon* (MIPS v1.2), rice (MSU v5) and Sorghum (MIPS v1.4) with functional annotation being extracted and recorded for each hit.

A linkage map of bread wheat, based on the transcriptome SNP markers, was constructed, essentially as described previously for the oilseed rape transcriptome SNP‐based linkage map (Bancroft *et al*., 2011). After scoring polymorphisms across a population of 47 single‐seed descent lines from a Chinese Spring × Paragon cross plus the parent lines, the markers were filtered in order to retain only those with BLAST hits to Brachypodium gene models. As the number of markers was too large to be processed by conventional linkage mapping software, the linkage map was constructed using string‐matching in MS Excel spreadsheets to first identify SNP markers with similar scoring strings (and therefore grouped close to each other in the genome) from a pool of unmapped markers. The clusters of markers identified were then ordered based on the order of the orthologous *Brachypodium* gene models and runs of collinear markers incorporated into recombination bins (624 in all) comprising sets of markers with identical allele calls. Noncollinear markers returned to the unmapped pool and the process repeated iteratively until the whole genome was covered. Unlinked blocks of markers were positioned relative to each other based on published collinearity analyses between the genomes of Brachypodium and wheat (International Brachypodium Initiative, 2010). A graphical genotype was generated and inspected manually to ensure that there were no inconsistencies that might indicate false assembly of the map.

Based on the BLAST hits of the unigenes containing the distal SNP markers in each block of collinearity between the wheat SNP map and the Brachypodium genome sequence, the Brachypodium genome sequence was split into segments, with the division mid‐way between the BLAST HSP coordinates defining the ends of rearranged blocks. These blocks were then re‐ordered and re‐oriented, based on their span of recombination bins in the linkage map, to establish a set of pseudomolecules representing the hypothetical organization of the orthologous sequences in the wheat genome, essentially as described previously for the *B. napus* pseudomolecules (Harper *et al*., 2012). Finally, the Trinity unigenes were aligned against the constructed pseudomolecules using BLASTN with an *E*‐value threshold of 1E‐30 and the chromosome and coordinates recorded for each best hit.

### Assessment of quantitative genome contributions to the transcriptome

To assess regions of genome dominance, redundant transcript assemblies were filtered in cases where multiple unigenes with the same Brachypodium BLAST hit were found with the same location on the genome. In this case, only the longest unigene was selected, reducing the number of unigenes to 147 411, comprising 47 160, 59 663 and 40 588 in the A, B and D genomes, respectively. Homoeologues were identified as unigenes in all three genomes with top reciprocal BLAST hits to each other (threshold *E*‐value 1E‐30). A total of 15 527 triplets of homoeologues were identified in this way. mRNA‐Seq reads from the wheat progenitor species and 54 varieties from the hexaploid wheat diversity panel were then mapped to the nonredundant 147 411‐unigene reference sequence and, using methods and scripts described in Bancroft *et al*. (2011) and Higgins *et al*. (2012), expression of each unigenes was estimated for each accession. Transcript abundance was quantified and normalized as reads per kb per million aligned reads (RPKM) separately for each unigene. Filtered data for the 15 527 triplets of homoeologues were retrieved for further analysis. Mapping statistics and average transcript abundance for each accession are provided in Data S9.

To identify homoeologues that may be differentially expressed across the genomes, a Tukey test was applied to each pair within a homoeologue triplet (i.e. AB, AD, BD). Where two of these pairwise tests (e.g. AB and AD) were significant (*P* < 0.05), the genome contributing to both (i.e. A) was defined as being differentially expressed. The mean RPKM values across the diversity panel were then compared to define relative up‐ or down‐regulation of that genome orthologue. Cases where a single test, or all three pairwise tests were significant, were ignored. All homoeologues were then placed in genome order and significant results colour‐coded as seen in Figure S6.

To identify expression structure in the genomes, a normalized tile plot was created. As the reference is based on diploid species representing the hexaploid genome progenitors, all with different evolutionary distances from bread wheat, it is reasonable to assume that there will be unequal efficiency of read mapping to the three genomes. To counteract this, a normalization based on the total number of mapped reads to each genome was used. RPKM values for each of the putative homoeologues were adjusted to a range between 1 and 0, where 1 is the individual with the lowest and 0 the individual with the highest expression value across the diversity panel. These values were then converted to RGB hexcodes and arranged in genome order to create a tile plot where the colour of each tile is converted corresponding to the given intensities of the red, green and blue primaries. According the standard CMYK colour space, if cyan, magenta and yellow intensities are equal, the resulting tiles will be a shade of grey, where boundaries are black CMY(1,1,1) and white CMY(0,0,0). A nongreyscale colour will show an expression difference between the homoeologous unigenes on the A, B and D genomes.

### Validation by cDNA sequencing

As an example, a triplet of homoeologues from a region identified as having increased A genome expression on linkage group 2 was selected for validation of relative expression, based on sequencing of cDNA. PCR primers were designed to amplify the set of homoeologues (A_comp34236_c0_seq1, B_comp4776_c0_seq1 and D_comp10830_c0_seq1) from cDNA (349 bp). Primer sequences were as follows: forward, GATGTATCAAGTTCTGCTCTTC; reverse, CTTATAGTGTCACCACCAATAAC. Capillary sequencing was then performed using the reverse primer to assess relative expression based on peak heights of IHPs, as measured using the Softgenetics Mutation Surveyor software. This approach was used for all further instances of validation of transcript abundance differences identified on the basis of mRNA‐Seq data.

### Comparison of triplets to wheat genome data

A list of 8605 homoeologous triplets (http://wheat-urgi.versailles.inra.fr/; IWGSC, 2014) based on wheat genome data were compared to our triplets following concatenation of splice variants to construct a single full‐length transcript for each gene. Reciprocal BLASTn (*E*‐value <1E‐30) was used to assess significant similarity between the sets of triplets. Using the same BLAST parameters, our homoeologous triplets were also compared to the full set of transcript sequences for all high‐confidence (HCS) gene models with a home on the IWGSC sequence assembly (excluding splice variants) (https://urgi.versailles.inra.fr), as these are likely to represent the approximate gene complement of the bread wheat subgenomes.

## Supporting information


**Figure S1** Collinearity of wheat pseudomolecule and 3B genome assembly.
**Figure S2** Collinearity between inferred gene order and the V5 wheat genome zipper for the A, B and D genomes.
**Figure S3** Tile plots.
**Figure S4** Tukey plots.
**Figure S5** Dot histogram.
**Figure S6** Homoeologue alignment.
**Figure S7** Inter‐homoeologue polymorphisms visualized by capillary sequencing.
**Figure S8** Workflow diagram for visualising homoeologue expression patterns.

**Data S1** Annotation of unigenes.

**Data S2** Transcriptome SNP linkage map for hexaploid wheat.

**Data S3** Consensus recombination bins representing the wheat SNP linkage map.

**Data S4** Collinearity of wheat SNP linkage map and Brachypodium genome.

**Data S5** Pseudomolecule specification.

**Data S6** Mapping of unigenes to pseudomolecules.

**Data S7** Significant Tukey tests.

**Data S8** Genome dominance validation.

Mapping statistics.
