# Supplementary material for: Genome distribution of differential homoeologue contributions to leaf gene expression in bread wheat
Source: Plant Biotechnol J. 2015 Oct 7;14(5):1207–14. doi: 10.1111/pbi.12486 (PMC4973816; doi:10.1111/pbi.12486)
Supplement: Supplementary file 1 — Figure S1 Collinearity of wheat pseudomolecule and 3B genome assembly. Figure S2 Collinearity between inferred gene order and the V5 wheat genome zipper for the A, B and D genomes. Figure S3 Tile plots. Figure S4 Tukey plots. Figure S5 Dot histogram. Figure S6 Homoeologue alignment. Figure S7 Inter‐homoeologue polymorphisms visualized by capillary sequencing. Figure S8 Workflow diagram for visualising homoeologue expression patterns. [file PBI-14-1207-s009.pdf]

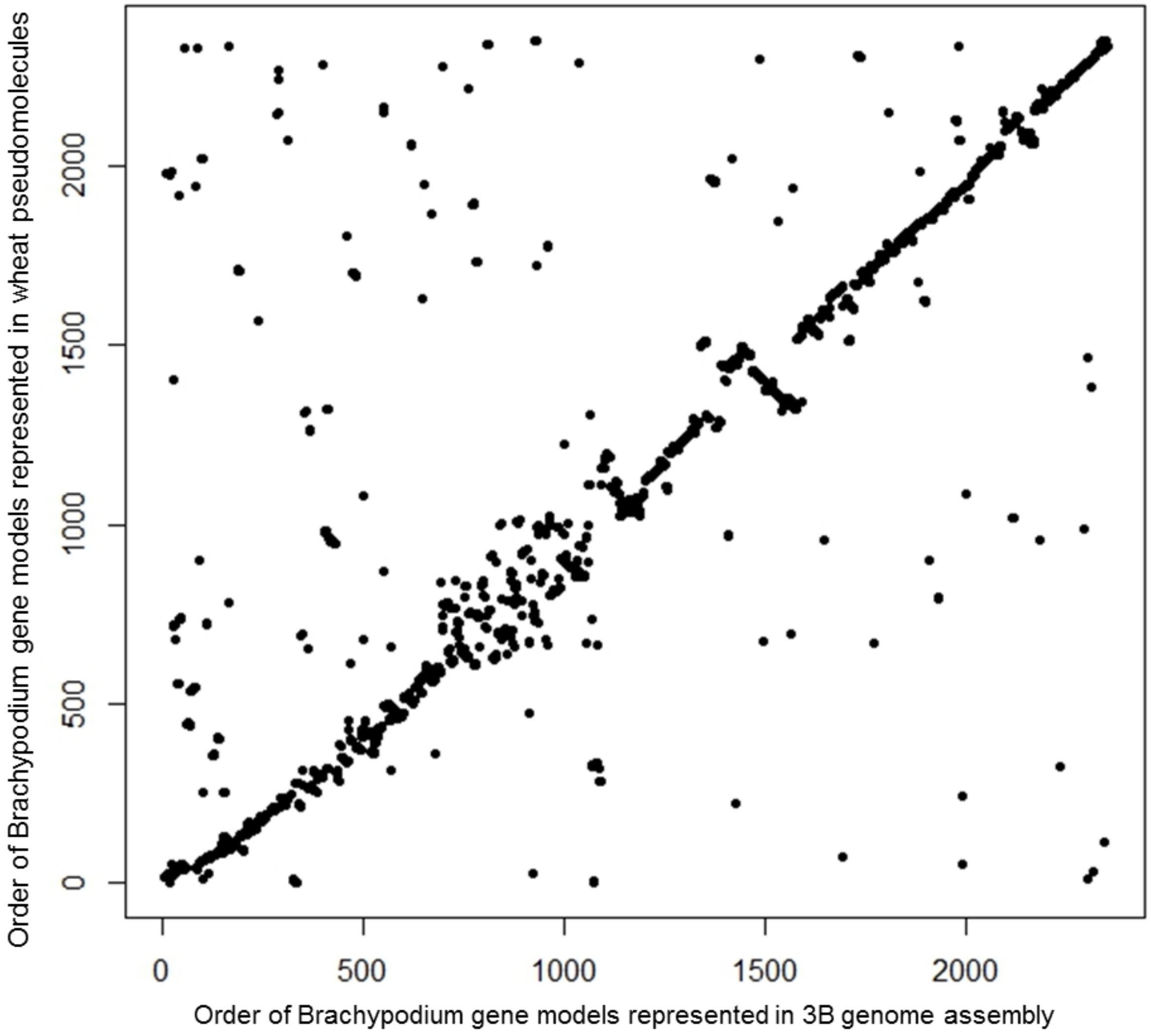

Supplementary Figure 1. Collinearity of wheat pseudomolecule and 3B genome assembly. Plot shows the relative positions of the 1,259 Brachypodium gene models represented in both the wheat pseudomolecules and the 3B genome assembly

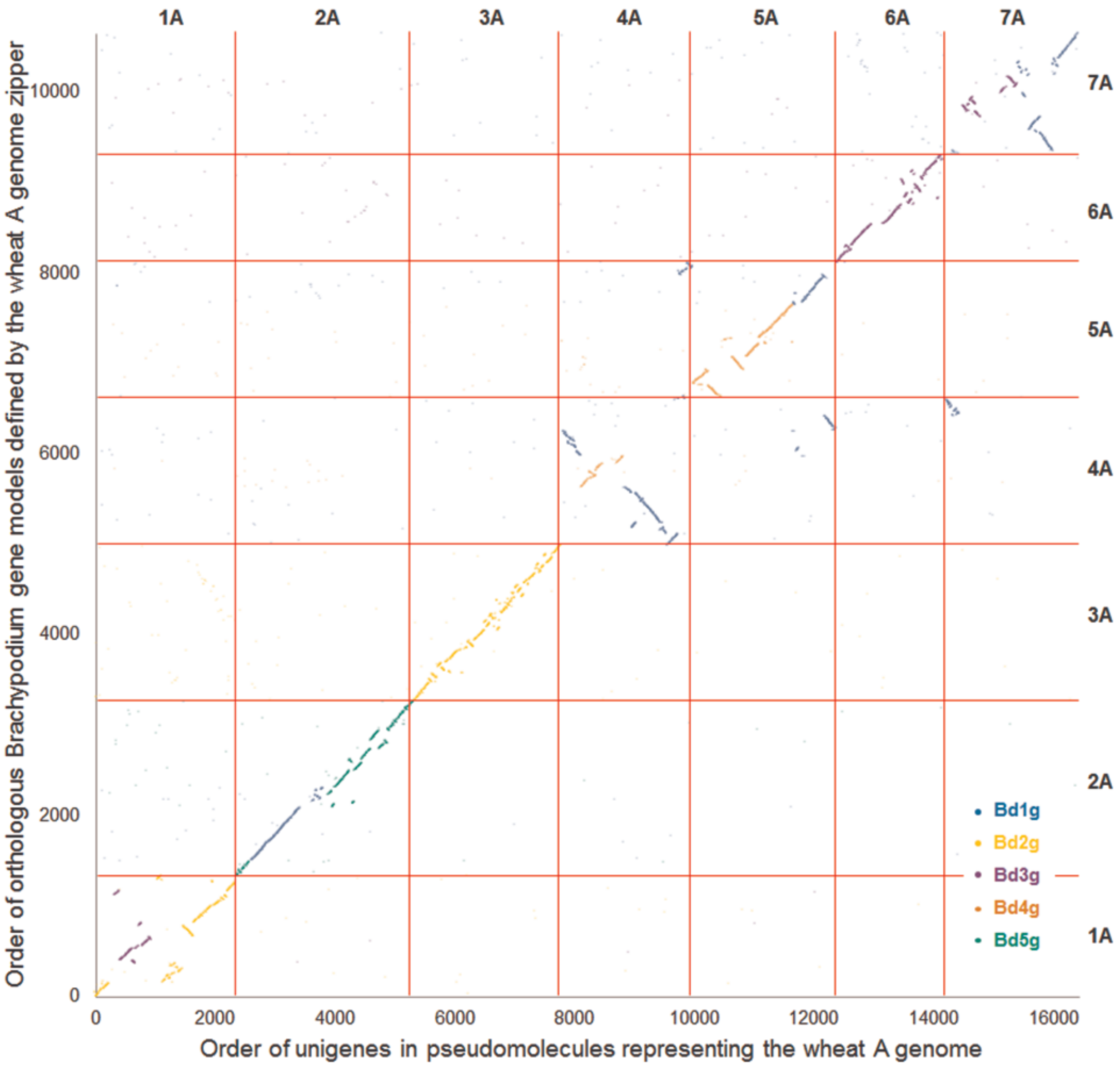

1A
2A
3A
4A
5A
6A
7A
Order of orthologous Brachypodium gene models defined by the wheat A genome zipper
0
2000
4000
6000
8000
10000
7A
6A
5A
4A
3A
2A
1A
Bd1g
Bd2g
Bd3g
Bd4g
Bd5g
0
2000
4000
6000
8000
10000
12000
14000
16000
Order of unigenes in pseudomolecules representing the wheat A genome

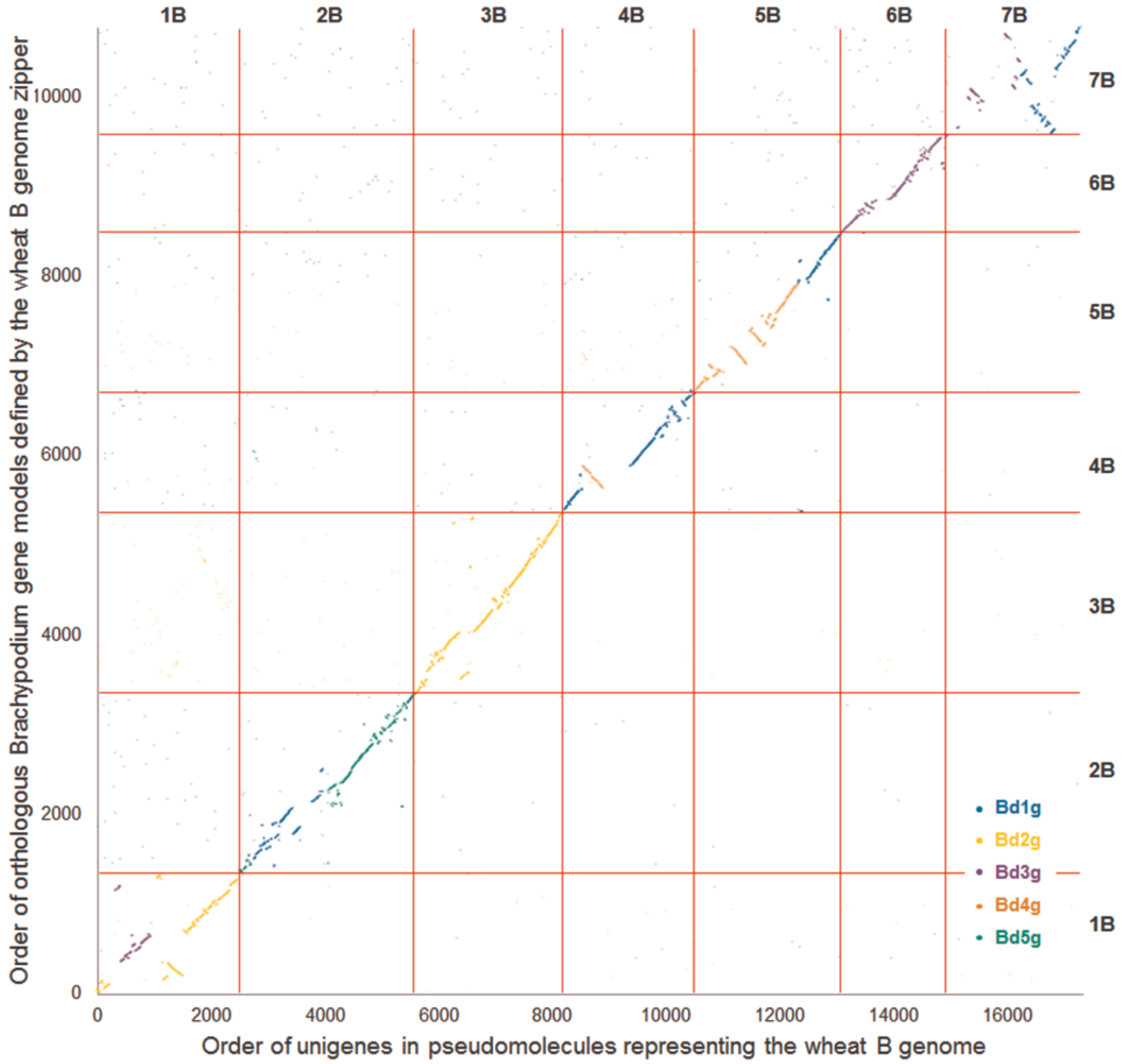
1B
2B
3B
4B
5B
6B
7B
Order of orthologous Brachypodium gene models defined by the wheat B genome zipper
Order of unigenes in pseudomolecules representing the wheat B genome
0
2000
4000
6000
8000
10000
12000
14000
16000
Bd1g
Bd2g
Bd3g
Bd4g
Bd5g

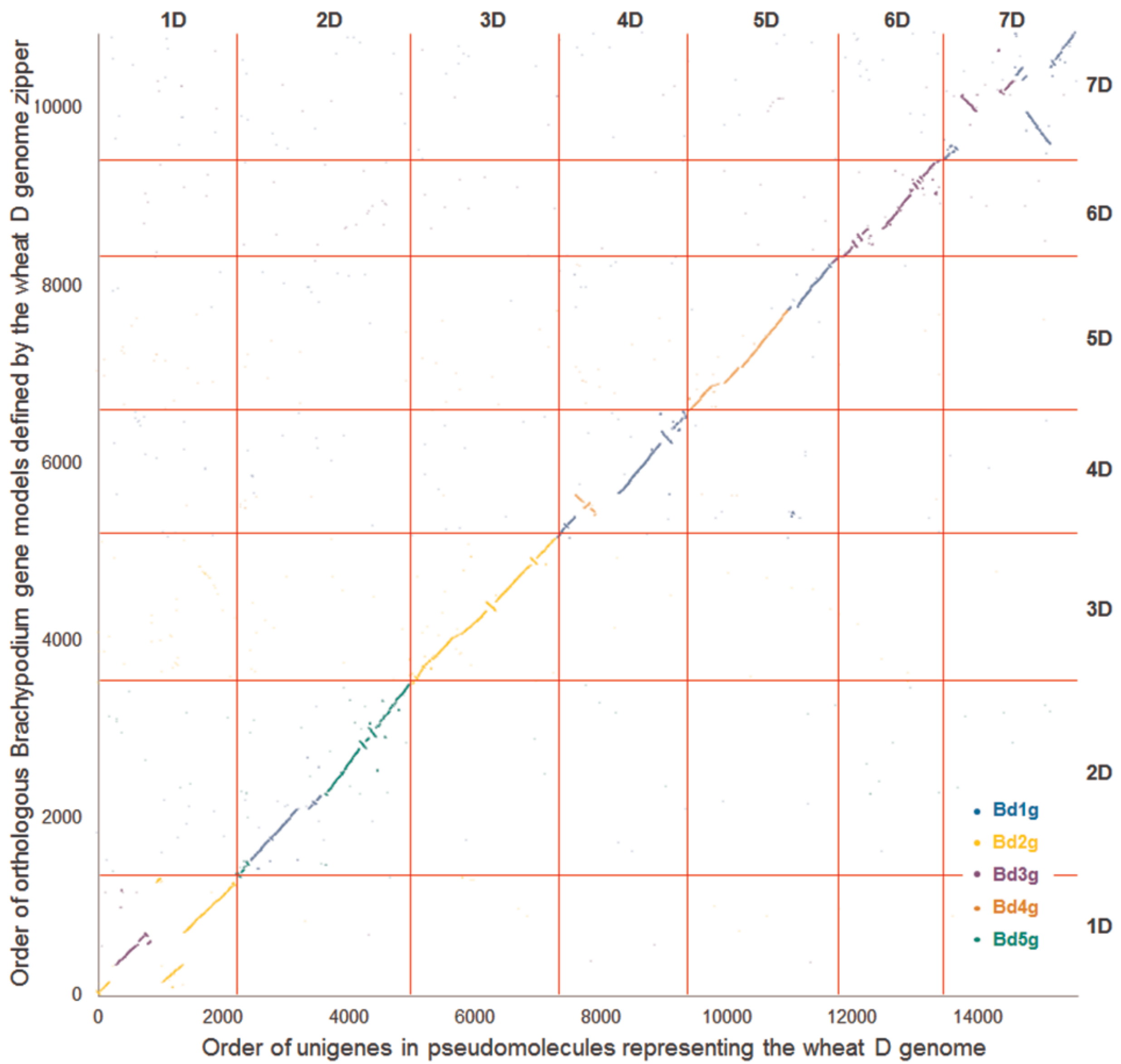

Supplementary Figure 2

Collinearity between inferred gene order and the V5 wheat genome zipper for the A B and D genomes. The plots show the order of 21,483 wheat unigenes in total with points color-coded by sequence similarity to the chromosome assignment of Brachypodium gene models: blue for chromosome 1, orange for chromosome 2, purple for chromosome 3, brown for chromosome 4 and green for chromosome 5

1

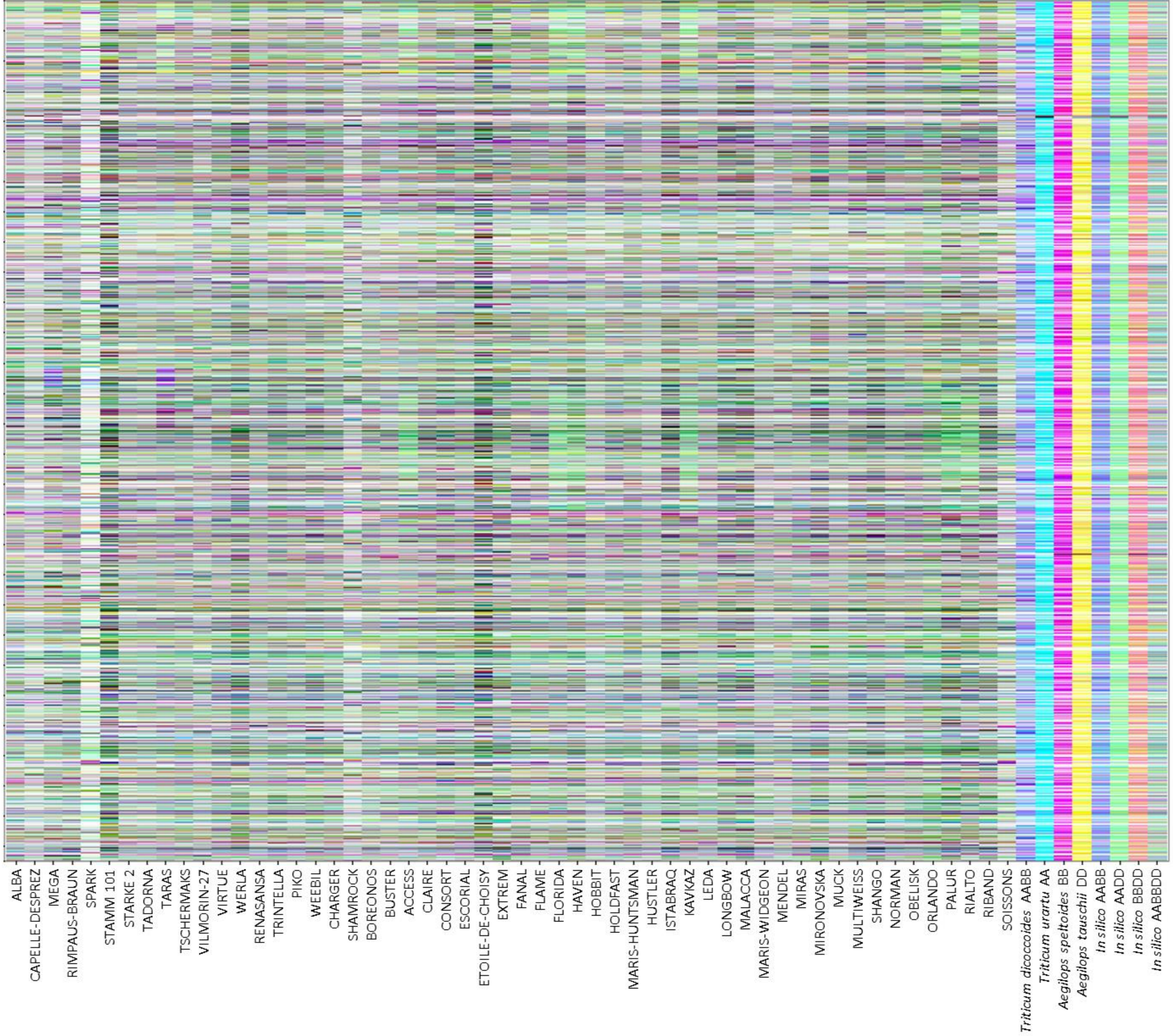
ALBA
CAPELLE-DESPREZ
MEGA
RIMPAUS-BRAUN
SPARK
STAMM 101
STARKE 2
TADORNA
TARAS
TSCHERMAKS
VILMORIN-27
VIRTUE
WERLA
RENASANSA
TRINTELLA
PIKO
WEEBIL
CHARGER
SHAMROCK
BOREONOS
BUSTER
ACCESS
CLAIRE
CONSORT
ESCORIAL
ETOILE-DE-CHOISY
EXTREM
FANAL
FLAME
FLORIDA
HAVEN
HOBBIT
HOLDFAST
MARIS-HUNTSMAN
HUSTLER
ISTABRAQ
KAVKAZ
LEDA
LONGBOW
MALACCA
MARIS-WIDGEON
MENDEL
MIRAS
MIRONOVSKA
MUCK
MULTIWEISS
SHANGO
NORMAN
OBELISK
ORLANDO
PALUR
RIALTO
RIBAND
SOISSONS
Triticum dicoccoides AABB
Triticum urartu AA
Aegilops speltoides BB
Aegilops tauschii DD
In silico AABB
In silico AADD
In silico BBDD
In silico AABBDD

2

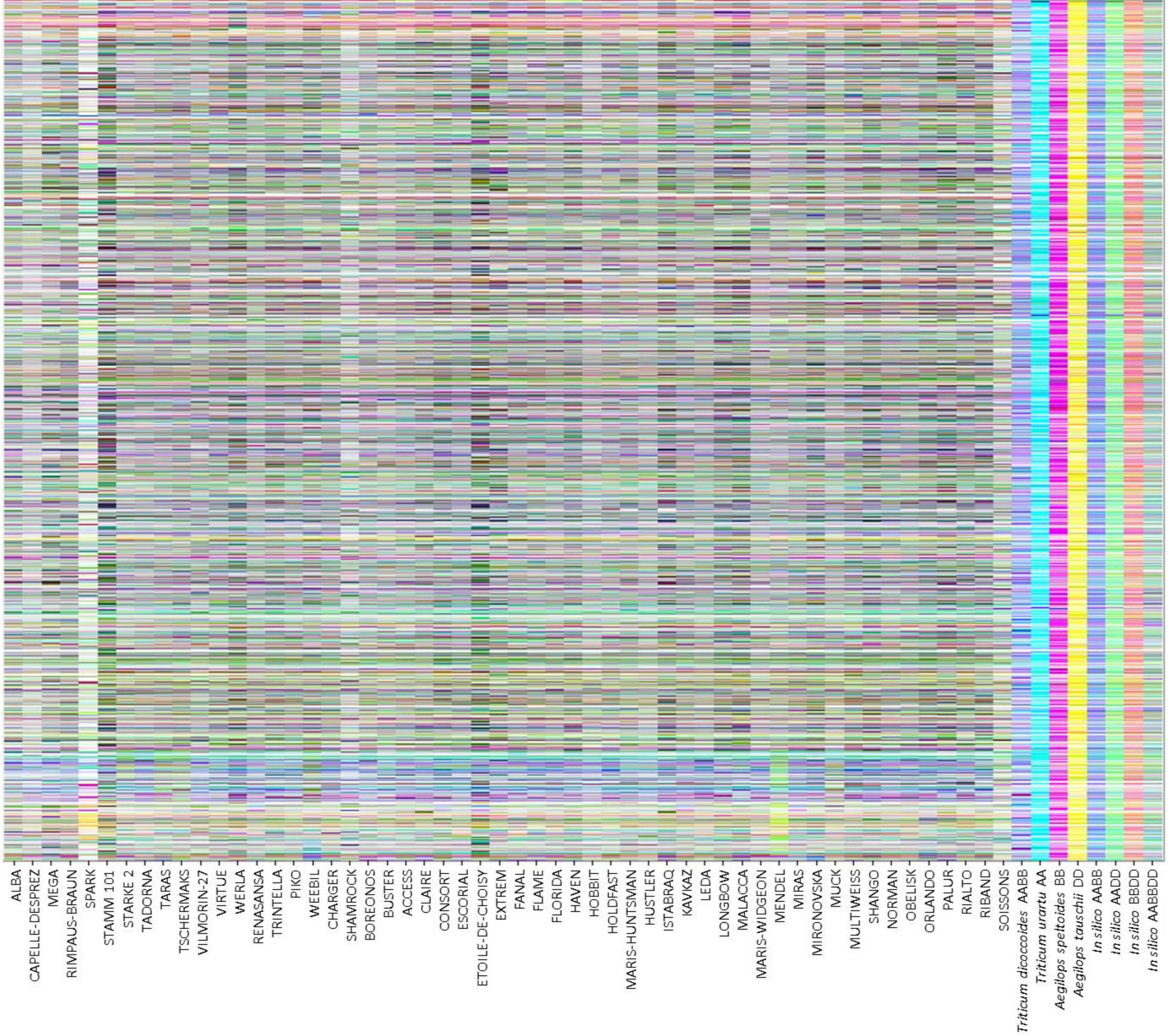
ALBA
CAPELLE-DESPREZ
MEGA
RIMPAUS-BRAUN
SPARK
STAMM 101
STARKE 2
TADORNA
TARAS
TSCHERMAKS
VILMORIN-27
VIRTUE
WERLA
RENASANSA
TRINTELLA
PIKO
WEEBIL
CHARGER
SHAMROCK
BOREONOS
BUSTER
ACCESS
CLAIRE
CONSORT
ESCORIAL
ETOILE-DE-CHOISY
EXTREM
FANAL
FLAME
FLORIDA
HAVEN
HOBBIT
HOLDFAST
MARIS-HUNTSMAN
HUSTLER
ISTABRAQ
KAVKAZ
LEDA
LONGBOW
MALACCA
MARIS-WIDGEON
MENDEL
MIRAS
MIRONOVSKA
MUCK
MULTIWEISS
SHANGO
NORMAN
OBELISK
ORLANDO
PALUR
RIALTO
RIBAND
SOISSONS
Triticum dicoccoides AABB
Triticum urartu AA
Aegilops speltoides BB
Aegilops tauschii DD
In silico AABB
In silico AADD
In silico BBDD
In silico AABBDD

3

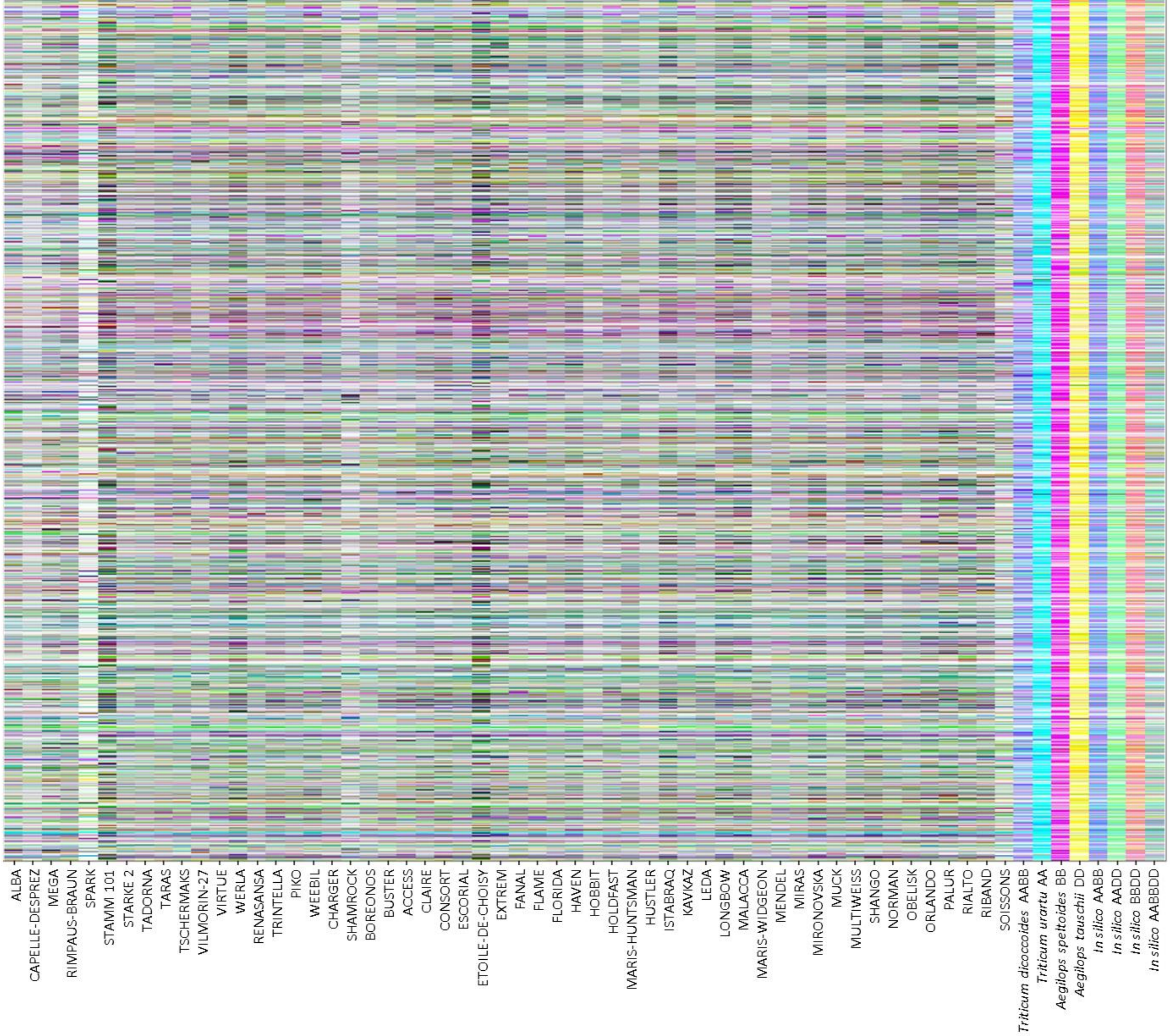
ALBA
CAPELLE-DESPREZ
MEGA
RIMPAUS-BRAUN
SPARK
STAMM 101
STARKE 2
TADORNA
TARAS
TSCHERMAKS
VILMORIN-27
VIRTUE
WERLA
RENASANSA
TRINTELLA
PIKO
WEEBIL
CHARGER
SHAMROCK
BOREONOS
BUSTER
ACCESS
CLAIRE
CONSORT
ESCORIAL
ETOILE-DE-CHOISY
EXTREM
FANAL
FLAME
FLORIDA
HAVEN
HOBBIT
HOLDFAST
MARIS-HUNTSMAN
HUSTLER
ISTABRAQ
KAVKAZ
LEDA
LONGBOW
MALACCA
MARIS-WIDGEON
MENDEL
MIRAS
MIRONOVSKA
MUCK
MULTIWEISS
SHANGO
NORMAN
OBELISK
ORLANDO
PALUR
RIALTO
RIBAND
SOISSONS
Triticum dicoccoides AABB
Triticum urartu AA
Aegilops speltoides BB
Aegilops tauschii DD
In silico AABB
In silico AADD
In silico BBDD
In silico AABBDD

4

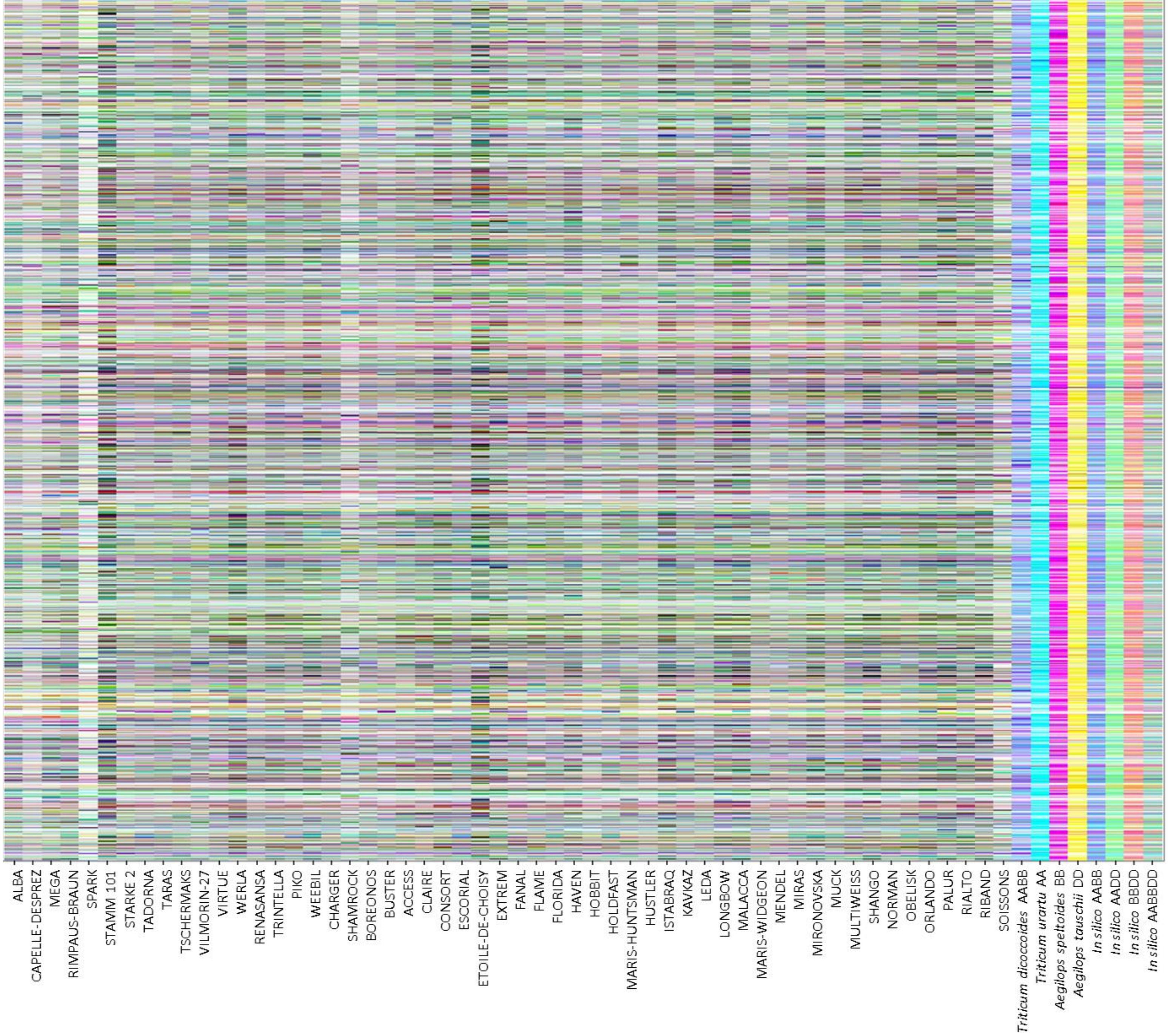
ALBA
CAPELLE-DESPREZ
MEGA
RIMPAUS-BRAUN
SPARK
STAMM 101
STARKE 2
TADORNA
TARAS
TSCHERMAKS
VILMORIN-27
VIRTUE
WERLA
RENASANSA
TRINTELLA
PIKO
WEEBIL
CHARGER
SHAMROCK
BOREONOS
BUSTER
ACCESS
CLAIRE
CONSORT
ESCORIAL
ETOILE-DE-CHOISY
EXTREM
FANAL
FLAME
FLORIDA
HAVEN
HOBBIT
HOLDFAST
MARIS-HUNTSMAN
HUSTLER
ISTABRAQ
KAVKAZ
LEDA
LONGBOW
MALACCA
MARIS-WIDGEON
MENDEL
MIRAS
MIRONOVSKA
MUCK
MULTIWEISS
SHANGO
NORMAN
OBELISK
ORLANDO
PALUR
RIALTO
RIBAND
SOISSONS
Triticum dicoccoides AABB
Triticum urartu AA
Aegilops speltoides BB
Aegilops tauschii DD
In silico AABB
In silico AADD
In silico BBDD
In silico AABBDD

5

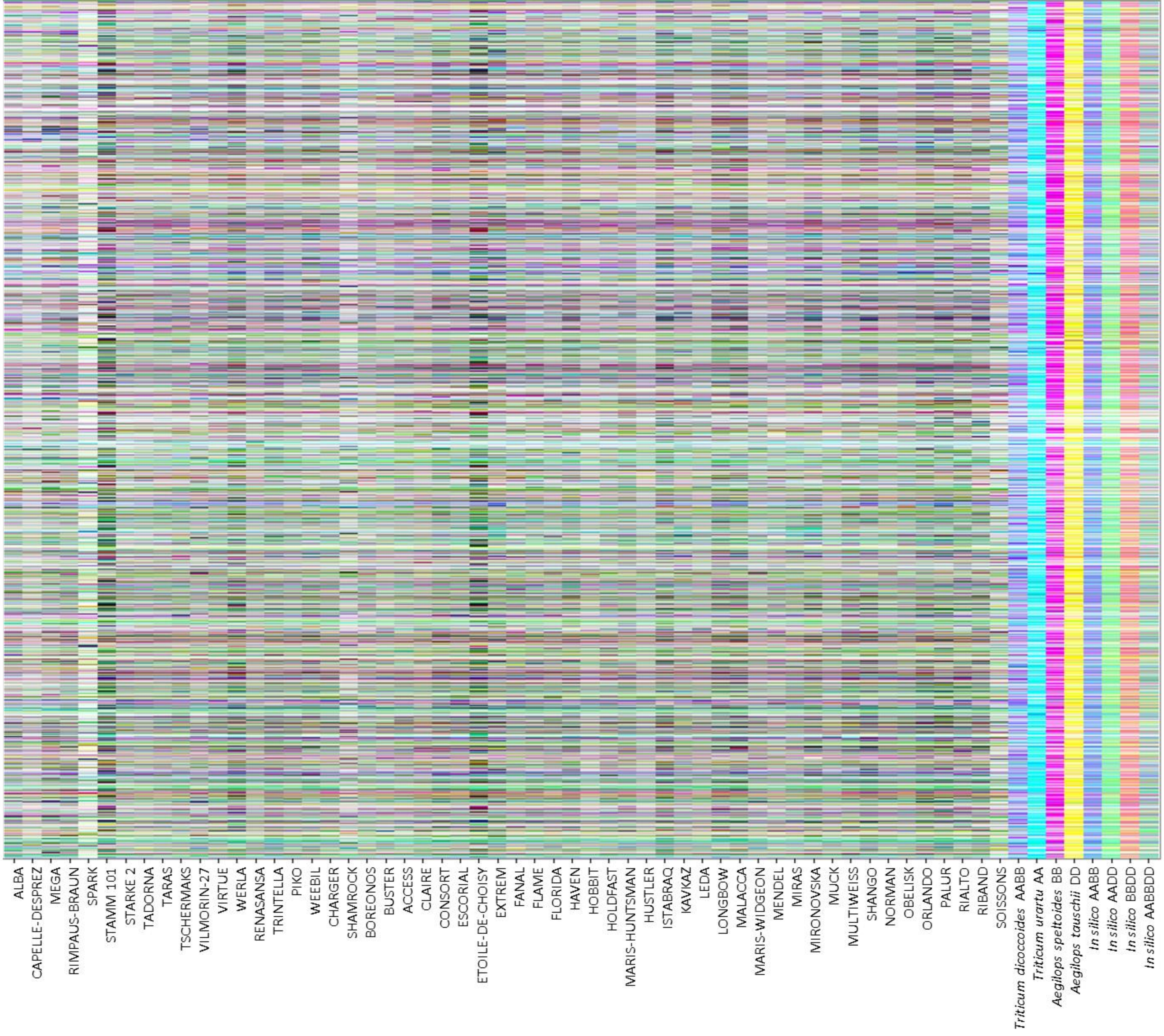
ALBA
CAPELLE-DESPREZ
MEGA
RIMPAUS-BRAUN
SPARK
STAMM 101
STARKE 2
TADORNA
TARAS
TSCHERMAKS
VILMORIN-27
VIRTUE
WERLA
RENASANSA
TRINTELLA
PIKO
WEEBIL
CHARGER
SHAMROCK
BOREONOS
BUSTER
ACCESS
CLAIRE
CONSORT
ESCORIAL
ETOILE-DE-CHOISY
EXTREM
FANAL
FLAME
FLORIDA
HAVEN
HOBBIT
HOLDFAST
MARIS-HUNTSMAN
HUSTLER
ISTABRAQ
KAVKAZ
LEDA
LONGBOW
MALACCA
MARIS-WIDGEON
MENDEL
MIRAS
MIRONOVSKA
MUCK
MULTIWEISS
SHANGO
NORMAN
OBELISK
ORLANDO
PALUR
RIALTO
RIBAND
SOISSONS
Triticum dicoccoides AABB
Triticum urartu AA
Aegilops speltoides BB
Aegilops tauschii DD
In silico AABB
In silico AADD
In silico BBDD
In silico AABBDD

6

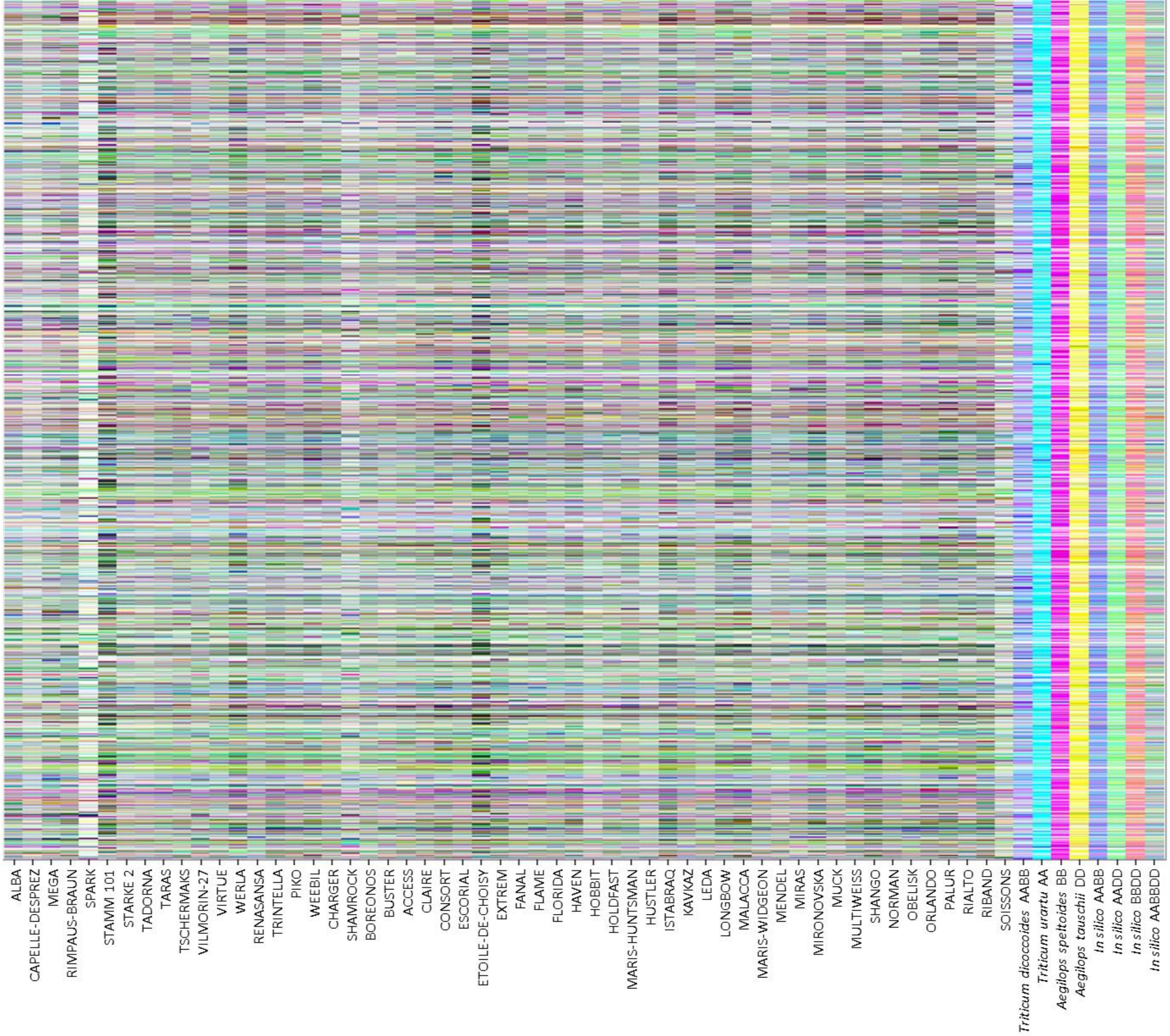
ALBA
CAPELLE-DESPREZ
MEGA
RIMPAUS-BRAUN
SPARK
STAMM 101
STARKE 2
TADORNA
TARAS
TSCHERMAKS
VILMORIN-27
VIRTUE
WERLA
RENASANSA
TRINTELLA
PIKO
WEEBIL
CHARGER
SHAMROCK
BOREONOS
BUSTER
ACCESS
CLAIRE
CONSORT
ESCORIAL
ETOILE-DE-CHOISY
EXTREM
FANAL
FLAME
FLORIDA
HAVEN
HOBBIT
HOLDFAST
MARIS-HUNTSMAN
HUSTLER
ISTABRAQ
KAVKAZ
LEDA
LONGBOW
MALACCA
MARIS-WIDGEON
MENDEL
MIRAS
MIRONOVSKA
MUCK
MULTIWEISS
SHANGO
NORMAN
OBELISK
ORLANDO
PALUR
RIALTO
RIBAND
SOISSONS
Triticum dicoccoides AABB
Triticum urartu AA
Aegilops speltoides BB
Aegilops tauschii DD
In silico AABB
In silico AADD
In silico BBDD
In silico AABBDD

7

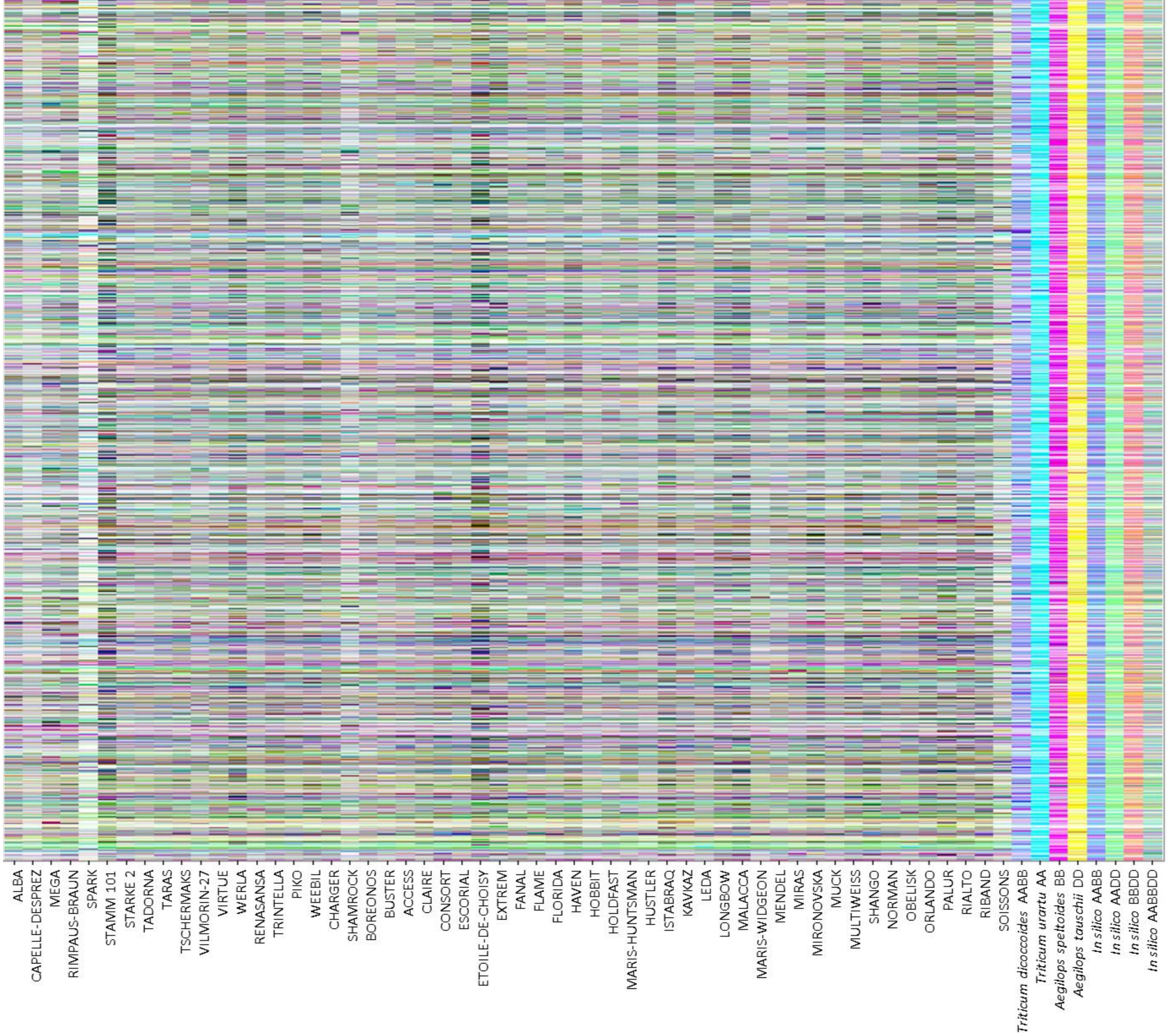
ALBA
CAPELLE-DESPREZ
MEGA
RIMPAUS-BRAUN
SPARK
STAMM 101
STARKE 2
TADORNA
TARAS
TSCHERMAKS
VILMORIN-27
VIRTUE
WERLA
RENASANSA
TRINTELLA
PIKO
WEEBIL
CHARGER
SHAMROCK
BOREONOS
BUSTER
ACCESS
CLAIRE
CONSORT
ESCORIAL
ETOILE-DE-CHOISY
EXTREM
FANAL
FLAME
FLORIDA
HAVEN
HOBBIT
HOLDFAST
MARIS-HUNTSMAN
HUSTLER
ISTABRAQ
KAVKAZ
LEDA
LONGBOW
MALACCA
MARIS-WIDGEON
MENDEL
MIRAS
MIRONOVSKA
MUCK
MULTIWEISS
SHANGO
NORMAN
OBELISK
ORLANDO
PALUR
RIALTO
RIBAND
SOISSONS
Triticum dicoccoides AABB
Triticum urartu AA
Aegilops speltoides BB
Aegilops tauschii DD
In silico AABB
In silico AADD
In silico BBDD
In silico AABBDD

Supplementary Figure 3. Genome contribution tile plots for the 7 bread wheat linkage groups. Normalised RPKM data from each triplet of 15,527 homoeologues in 54 bread wheat cultivars were visualised in pseudomolecule order as CMY tile plots where A genome only contributions produce cyan, B genome magenta and D genome yellow coloured cells, and equal contributions from all three genomes, grey cells. These were compared to the RPKM from transcriptome assemblies of the three representative diploids (AA, *Triticum urartu*; BB, *Aegilops speltoides*; DD, *Aegilops tauschii*), the AABB tetraploid *Triticum dicoccoides*, and equal tetraploid and hexaploid genome combinations produced *in silico*.

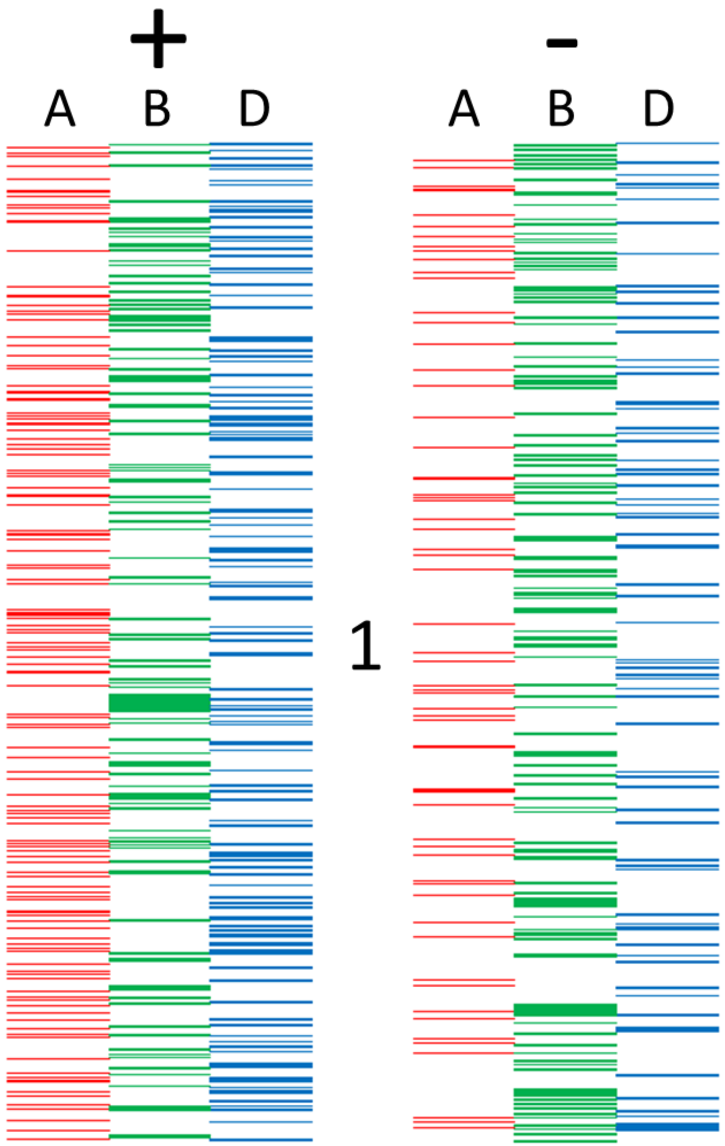
+
A B D
-
A B D
1

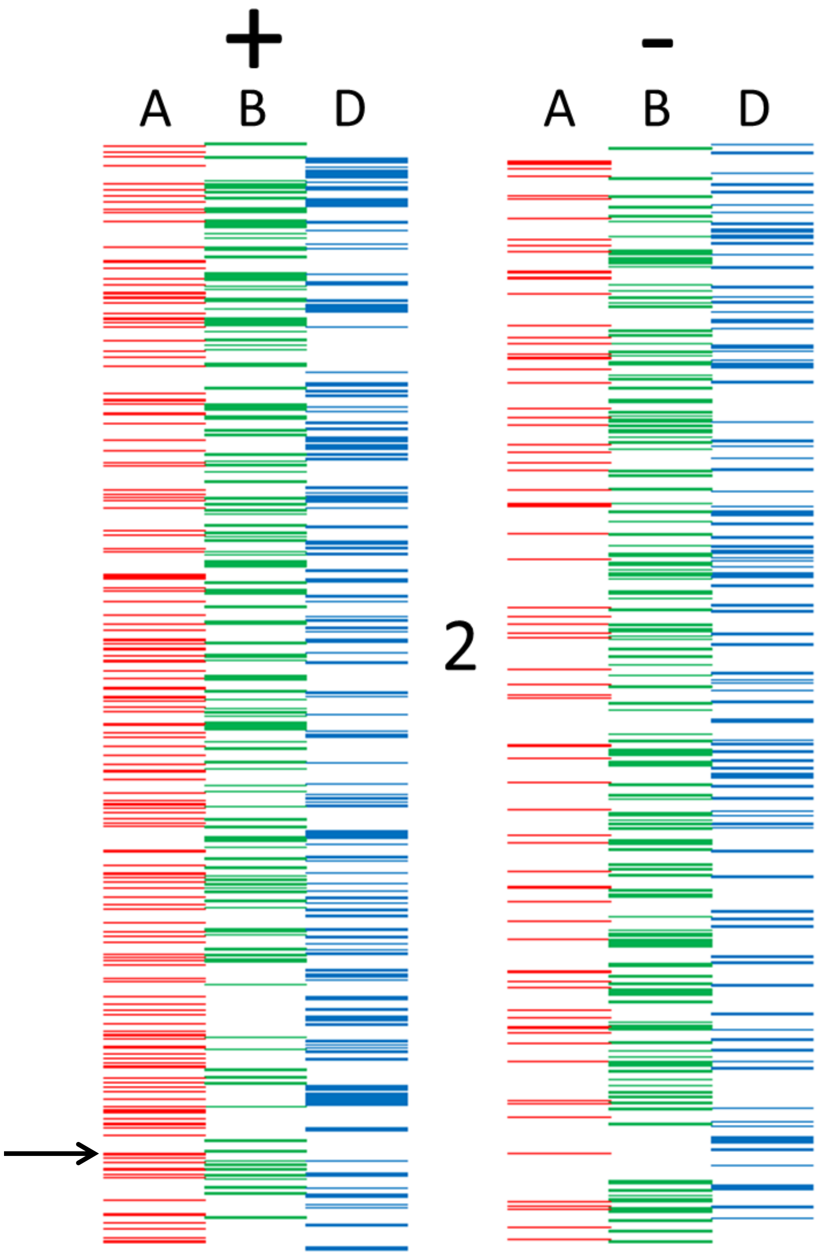

+
A
B
D
-
A
B
D
2

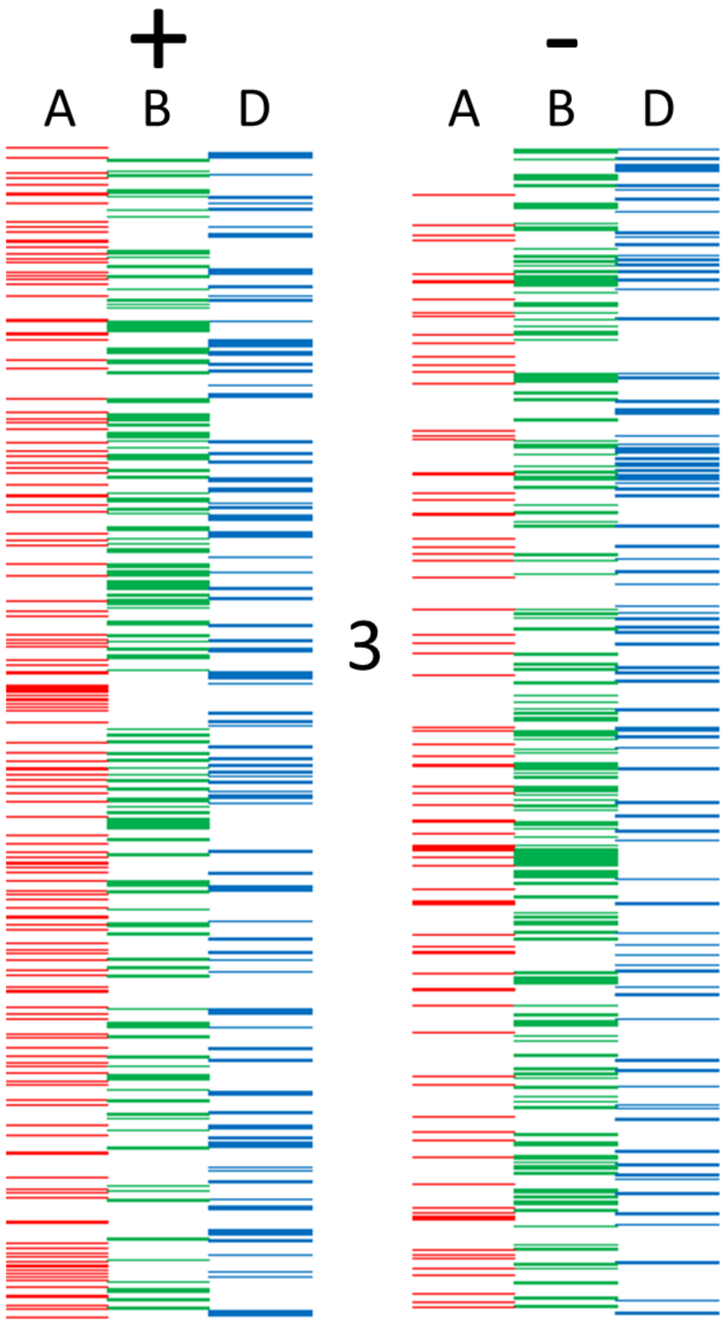

+
A
B
D
-
A
B
D
3

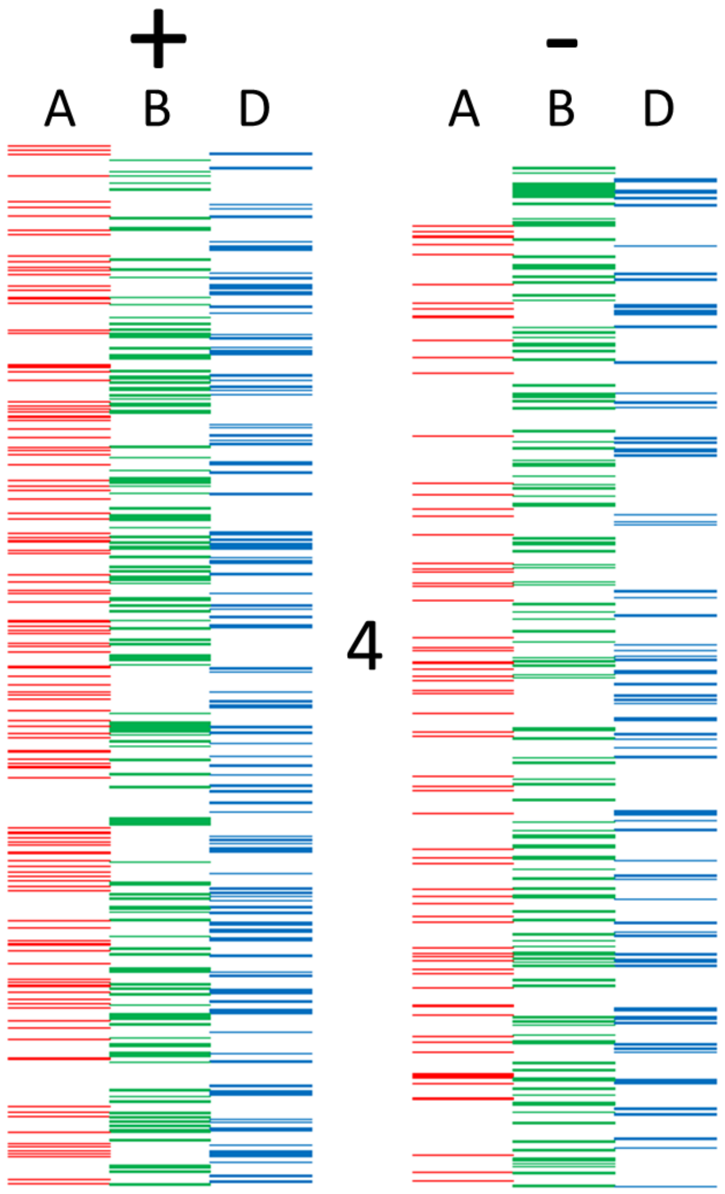
+
A B D
-
A B D
4

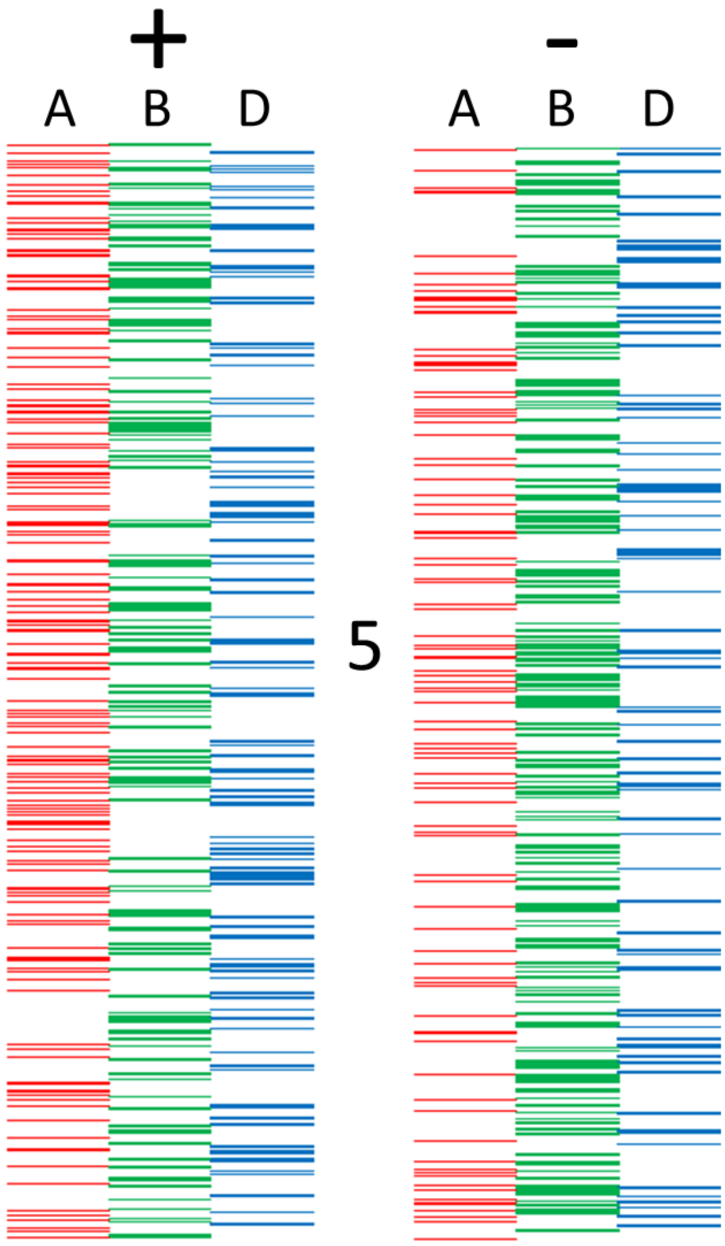
+
-
A
B
D
A
B
D
5

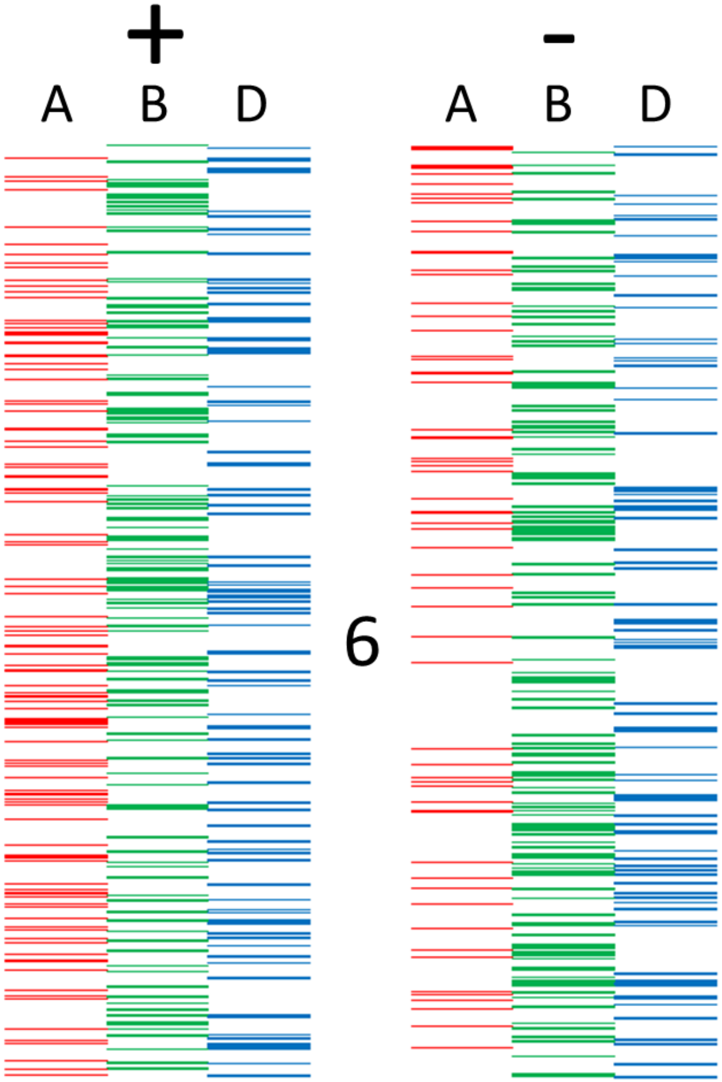
+
A
B
D
-
A
B
D
6

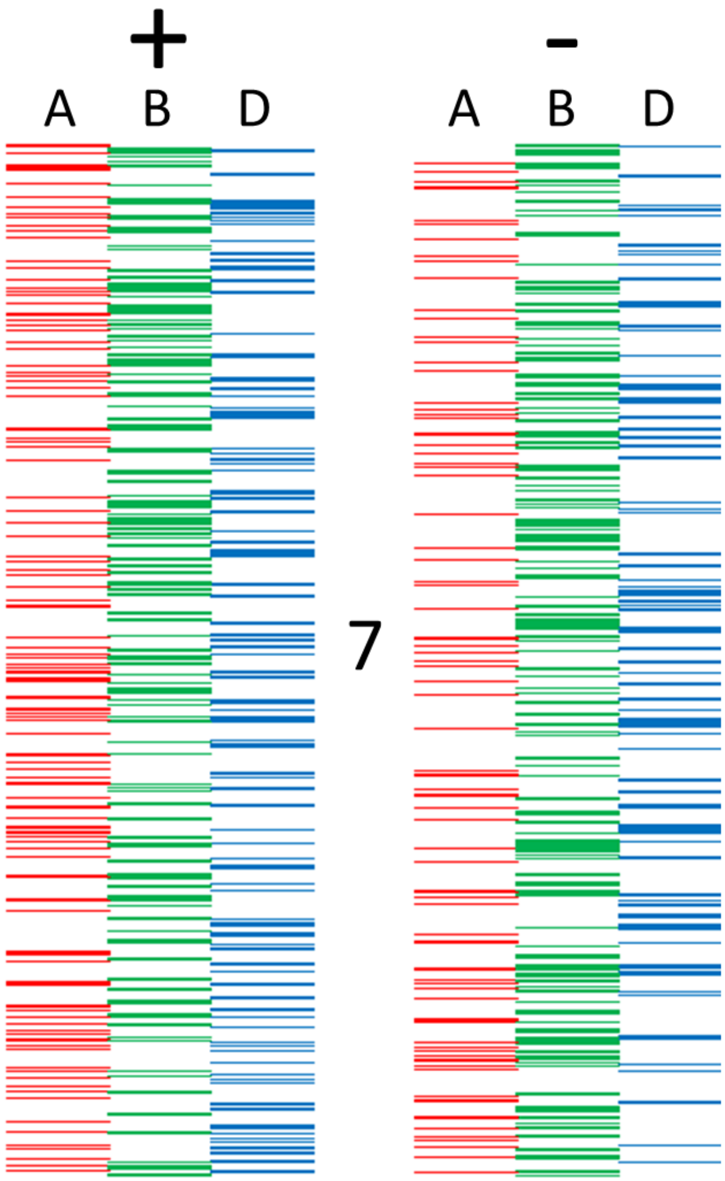

+
-
A
B
D
A
B
D
7

Supplementary Figure 4. Tukey tests for genome dominance. A Tukey test was performed to identify instances where normalised transcript abundance for a single genome homoeologue differs significantly from the abundance of the other two. Significantly differing homoeologues were then assigned as up- or down-regulated relative to the other genome, and plotted in separate channels in genome order. Red lines indicate the A genome, green lines the B genome and blue lines the D genome homoeologue. The arrow on linkage group 2 shows the location of the triplet used for validation.

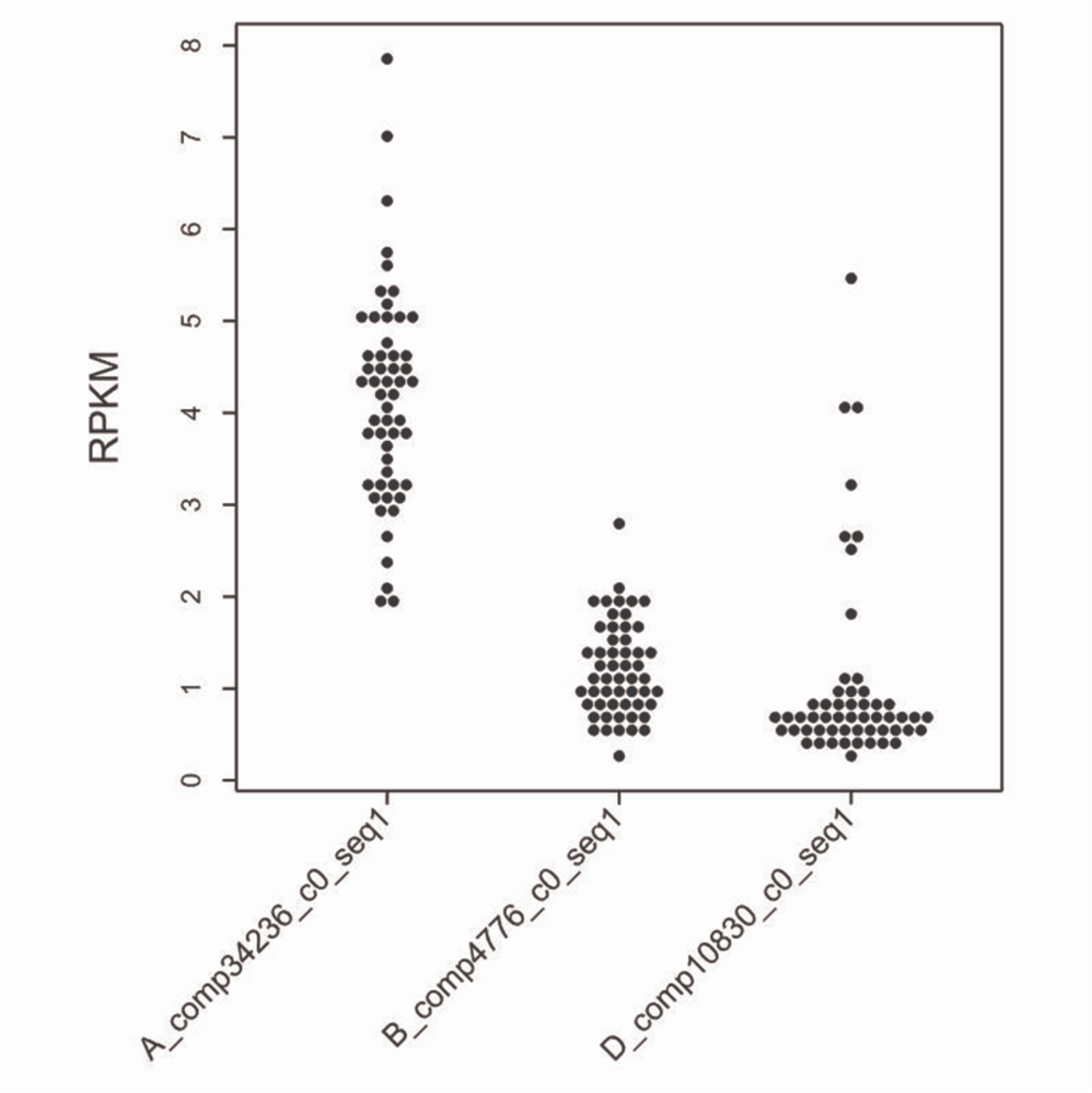

Supplementary Figure 5. Dot histogram showing RPKM values of 54 bread wheat accessions for a single set of A B and D homoeologues.

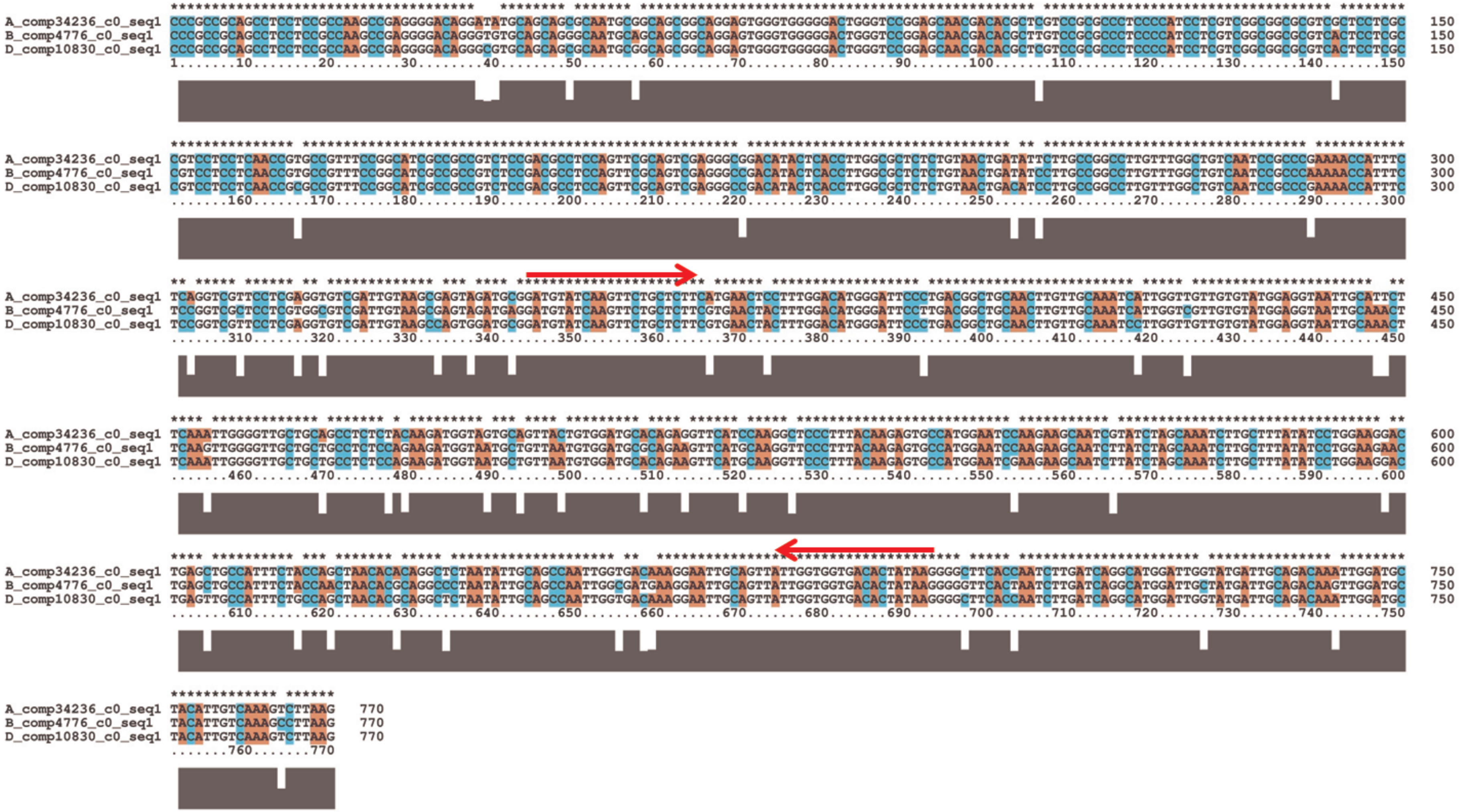

Supplementary Figure 6. Alignment of single set of A B and D homoeologues. Homoeologous transcripts were identified using 3-way reciprocal BLAST analysis and aligned using ClustalW. Identical positions are marked with asterisks. Non-identical positions are inter-homeologue polymorphisms (IHPs). Primer positions are marked by red arrows.

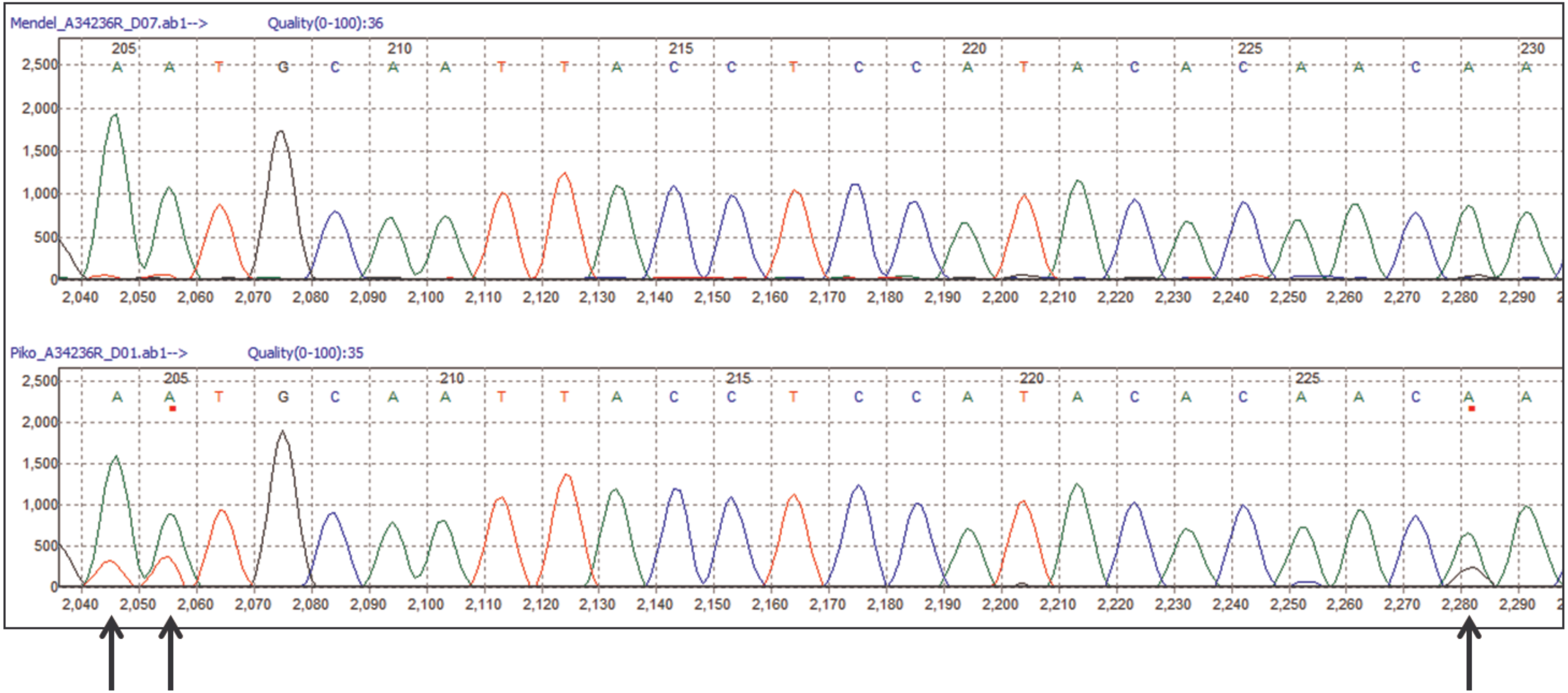

Supplementary Figure 7. Sequence chromatograms for two bread wheat accessions, Mendel and Piko, exhibiting expression variation at inter-homoeologue polymorphism (IHP) positions. IHP positions are marked with arrows.

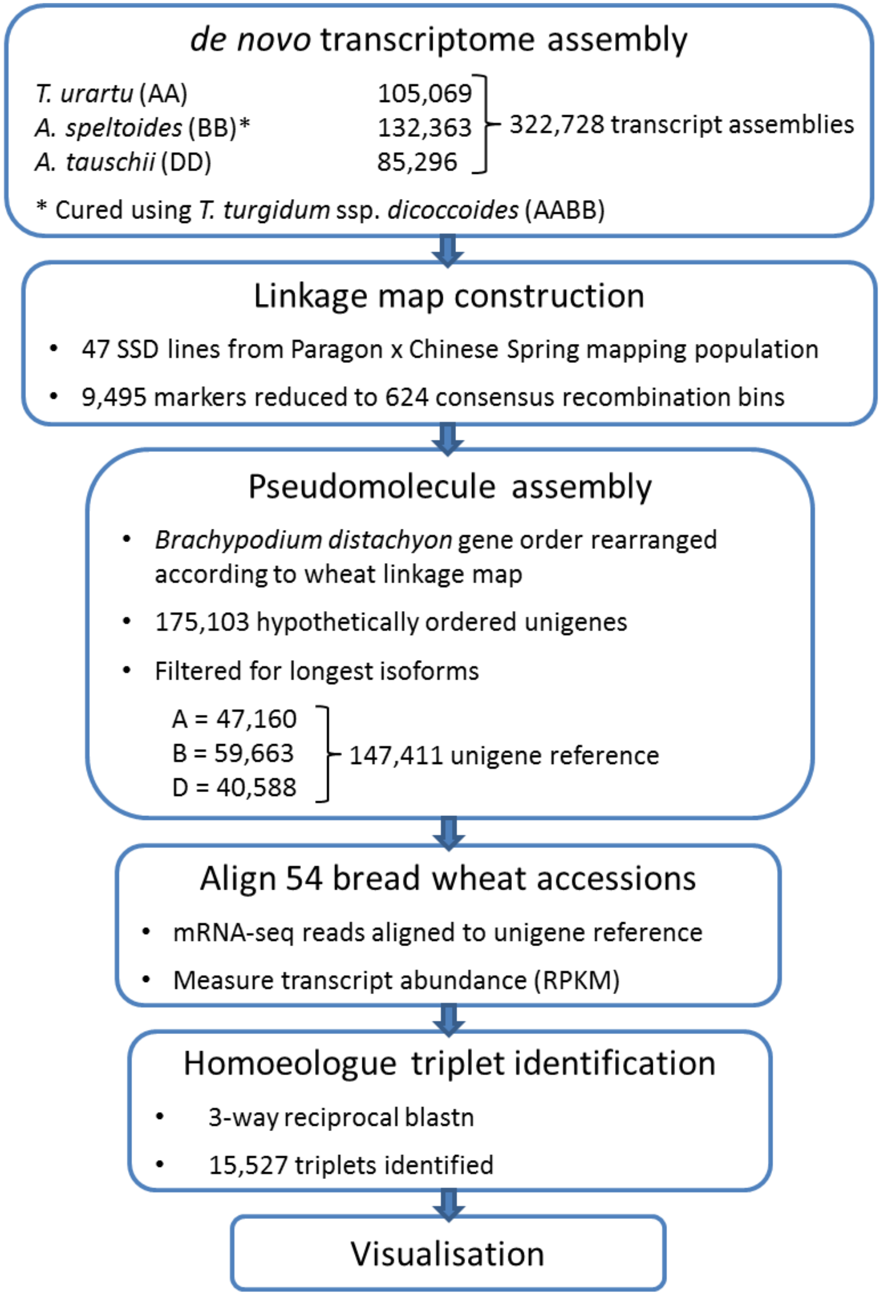

Supplementary Figure 8. Workflow diagram for visualising homoeologue expression patterns
